# Cell density-dependent antibiotic tolerance to inhibition of the elongation machinery requires fully functional PBP1B

**DOI:** 10.1038/s42003-022-03056-x

**Published:** 2022-02-03

**Authors:** Addison Grinnell, Ryan Sloan, Randy M. Morgenstein

**Affiliations:** Department of Microbiology and Molecular Genetics, Oklahoma State University, Stillwater, OK USA

**Keywords:** Antimicrobial resistance, Bacteriology, Cellular microbiology

## Abstract

The peptidoglycan (PG) cell wall provides shape and structure to most bacteria. There are two systems to build PG in rod shaped organisms: the elongasome and divisome, which are made up of many proteins including the essential MreB and PBP2, or FtsZ and PBP3, respectively. The elongasome is responsible for PG insertion during cell elongation, while the divisome is responsible for septal PG insertion during division. We found that the main elongasome proteins, MreB and PBP2, can be inhibited without affecting growth rate in a quorum sensing-independent density-dependent manner. Before cells reach a particular cell density, inhibition of the elongasome results in different physiological responses, including intracellular vesicle formation and an increase in cell size. This inhibition of MreB or PBP2 can be compensated for by the presence of the class A penicillin binding protein, PBP1B. Furthermore, we found this density-dependent growth resistance to be specific for elongasome inhibition and was consistent across multiple Gram-negative rods, providing new areas of research into antibiotic treatment.

## Abstract (web summary)

Grinnell et. al report a new mechanism of antibiotic tolerance in rod-shaped bacteria that is dependent on cell density and which specifically targets the cell wall elongation system. Their findings suggest that simultaneous disruption of both the elongasome and the divisome may be an efficient way of overcoming density-dependent growth resistance.

## Introduction

Bacterial cell shape is primarily maintained by the peptidoglycan cell wall, which is made up of sugar polymers crosslinked together by small peptides. Synthesis is catalyzed by a series of penicillin-binding proteins (PBPs) with transglycosylase and/or transpeptidase activity^1^. The two main classes of PBPs are class A PBPs (aPBP), which are bifunctional enzymes that can perform both transglycosylase and transpeptidase reactions, and class B PBPs (bPBPs), which are monofunctional transpeptidases. In many rod-shaped bacteria, cell wall synthesis is split among two homologous groups of proteins known as the divisome and the elongasome^2^. The divisome synthesizes and organizes septum synthesis and is coordinated by the tubulin homolog, FtsZ. The elongasome synthesizes and organizes side wall synthesis during growth and elongation and is coordinated by the bacterial actin homolog, MreB^1,3^. There is evidence that these two complexes interact with each other^2,4^.

The divisome complex is made up of an aPBP, PBP1B, and an essential bPBP, PBP3. PBP3 works in conjugation with FtsW, a SEDS (shape, elongation, division, sporulation) protein family member^5–7^. Interference of the divisome results in long filamentous cells as septation is disrupted^8,9^. The elongasome complex is made up of a homologous system to the divisome with an aPBP, PBP1A, and the essential bPBP, PBP2^2^. PBP2 works in conjugation with the transglycosylase, RodA, its SEDS protein family member^2^. The polymeric form of MreB directs sites of cell wall remodeling and coordinates the activity of the other members of the elongasome^1,10–12^. Current work suggests that MreB directly interacts with PBP2/RodA but is semi-autonomous from PBP1A^13–15^. The disruption of the elongasome results in dysregulated cell wall insertion during growth, resulting in swollen round cells^11^.

Interestingly, while disruption of MreB and PBP2 both result in similar round shape changes, they do not share similar overall physiology phenotypes^16,17^. MreB was discovered in screens for mutants resistant to the PBP2-targeting drug, mecillinam^18^. Antibiotics targeting PBPs, such as mecillinam and other beta-lactams, induce a futile cycle by both inhibiting new cell wall synthesis and inducing the breakdown of the current cell wall, while inhibition of MreB with the drug A22 does not induce the breakdown of the cell wall^16,19^.

MreB is conditionally essential and deletion can only be achieved by very slow growth or with the accumulation of suppressors, such as the upregulation of *ftsZAQ*. However, during the stationary phase, the transcriptional repressor BolA can repress *mreB* expression^20,21^. Ectopic expression of *bolA* during the exponential phase leads to a round cell phenotype similar to that seen when MreB is depolymerized by A22^22^. From this, we hypothesized that MreB might be dispensable and therefore less sensitive to A22 during the stationary phase when BolA should be most actively repressing its expression.

As expected, the addition of A22 to cells after 6 h of growth resulted in no change to growth rate; however, we found that A22 can be added as early as 2 h after inoculation without affecting the growth rate of cells. Here, we show that the effects of A22 on cell growth work in a quorum sensing (QS)-independent, cell density-dependent manner, while loss of cell shape is independent of cell density. Furthermore, this cell density-dependent growth resistance is only seen when the elongasome components MreB and PBP2 are targeted, but not other cell wall synthesis proteins. We show that the ability of cells to continue to grow as round cells requires the activity of the divisome protein PBP1B. Additionally, this cell density-dependent growth resistance (DDGR) appears in other MreB-containing Gram-negative rod species.

## Results

### Timing of A22 addition is important for growth rate changes

MreB is essential for rod shape in many bacteria. It is thought that MreB organizes the localization of the cell wall synthesis machinery; therefore, MreB should be at maximal expression and activity during the exponential phase when cells are growing the fastest. In *E. coli*, the transcription factor BolA is most active during the stationary phase^22^. It has been shown that BolA can repress transcription of MreB, contributing to the change in length:width ratio in stationary phase cells^13,23^. Therefore, we hypothesized that disrupting MreB with the depolymerizing agent A22 when cells are close to the stationary phase will have less of an inhibitory effect on cell growth than when cells are disrupted with A22 in the exponential phase^24^.

**Fig. 1:**
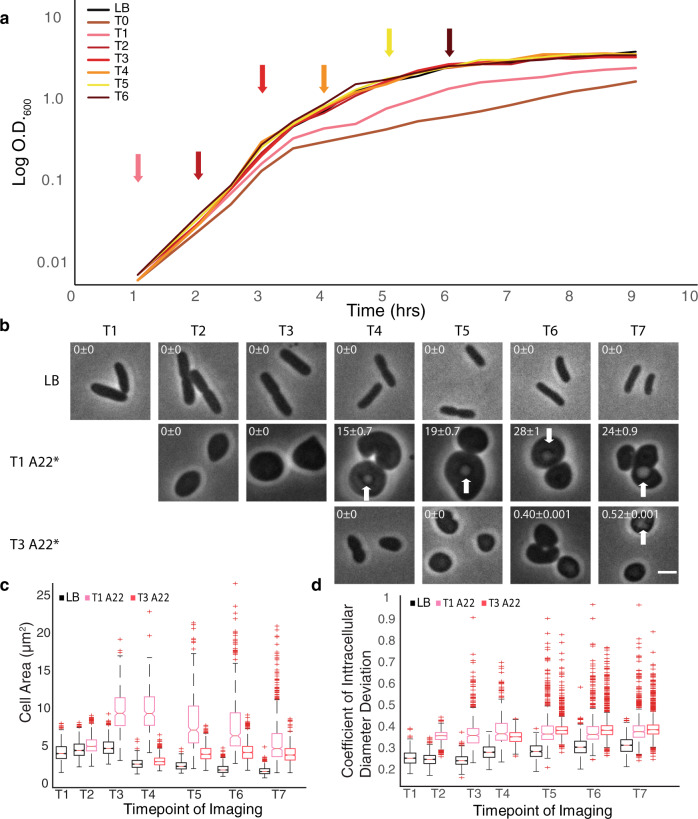
Timing of A22 addition is important for growth rate changes. **a** Representative growth curve of WT cells at 37 °C with A22 (10 µg/ml) added every hour from 0 to 6 h as indicated by corresponding arrows. **b** Representative phase-contrast images of WT cells grown at 37 °C in LB, LB with A22 (10 μg/ml) added at 1 h of growth, and LB with A22 added at 3 h at growth. Cells were imaged every hour for 7 h. White arrow indicates cell vesicles forming in advance of lysis and numbers in the upper left represent the percentage of cells displaying vesicles and the Poisson error to one standard deviation. The scale bar is equal to 2 µm. **c**, **d** Quantification of cells imaged in **b**. Red pluses are outliers, the red line is the median, the bottom and top edges of the box represent 25th and 75th percentiles, respectively, and notches are 95% CI. Data were pooled from three independent experiments. **c** Cell area from cells imaged in **b**. **d** Coefficient of intracellular diameter deviation from cells imaged in **b**. **c**, **d** Number of cells can be found in table S2.

To test our hypothesis, we performed growth curves with wild-type (WT) *E. coli* with the addition of A22 (10 µg/ml) every hour from T0–6 (Fig. 1a and S1). As we anticipated, the addition of A22 near the stationary phase (T5–6) showed little to no change in growth rate. Surprisingly, when A22 was added during the log phase, we observed little change in growth rate when compared with the LB only control (T2–4). Only cells still in the lag phase (T0–1) showed a marked difference in growth after the A22 addition. T3 will be used going forward to test this A22 inoculum time effect unless otherwise stated. Therefore, any differences in growth due to the time of inoculation are most likely independent of BolA as *bolA* is not observed to be expressed until T4^25^.

Disruption of MreB by A22 should lead to round cells^24,26^. We compared the cell shape characteristics of sensitive cells (T1) treated with A22 to resistant cells (T3) treated with A22. Cells were imaged every hour for 7 h with and without A22 added at T1 or T3 (Fig. 1b). To quantify the change in rod shape, we measured the intracellular diameter deviation (IDD); a measure of the standard deviation of the cell width across the centerline of each cell where a larger IDD indicates cells are less rod-shaped and more round^13,23^. Interestingly, both T1 and T3 A22 treated cells become equally round in roughly the same amount of time after treatment (Fig. 1c) suggesting there is no difference in the degree and speed of MreB depolymerization in sensitive or resistant cells.

Surprisingly, although both T1 and T3 treated cells become round after A22 addition, the size of the cells is different. The cell area of T1 treated cells more than doubles (4.30 to 9.82 µm^2^) within the first 2 h of A22 treatment, while T3 treated cells become slightly smaller (5.06 to 4.31 μm^2^) during that same length of A22 treatment (Fig. 1d). T1 treated cells do not grow during the time course as the cell area actually decreases (9.88 to 5.89 μm^2^); however, T3 treated cells show a slight increase in cell area (3.21 to 4.47 μm^2^) during the time course suggesting that these cells are still able to grow.

We also observed that cells treated with A22 at T1 commonly develop intracellular vesicles (Fig. 1b arrows) suggesting they are in the early stages of lysis^27^. We hypothesize this decrease in cell size seen in T1 treated cells is a result of larger cells lysing, leaving only smaller cells to measure. Unlike T1 treated cells, T3 treated cells rarely develop vesicles (0.4–0.5%). These data suggest there are physiological differences between cells treated with A22 at T1 versus T3. To determine if the phase-negative spots were indeed intracellular vesicles, transmission electron microscopy (TEM) of WT cells treated with A22 was performed. The phase-negative spots appear to be empty vesicles and not filled granules (Fig. S2a white arrows), although some contain what appear to be small portions of cytoplasm (Fig. S2a black arrow). The TEM also shows evidence of membrane shedding and possible bleb formation (Fig. S2a blue arrow) supporting a previous hypothesis that large round cells create an excess of the membrane that results in vesicle formation to shed the excess membrane^28,29^. Possible asymmetric division events were also observed (Fig. S2a red arrow). To determine if vesicle presence corresponded to cell death, we performed time-lapse imaging of WT cells presenting vesicles over the course of 8 h in the presence of A22 (Fig. S2b). Cells maintain their roundness and develop more vesicles over time before lysing.

### Growth resistance to A22 is due to cell density

Cells grow at normal rates when A22 is added at T3 but not T0/1. There are many factors that are different between the cells at these time points. First, cells are growing faster at T3 (log phase) than they are at T0 (lag phase). To test if the growth rate is responsible for the observed growth differences in A22, we grew cells at 30 ^o^C to slow down the growth rate. A22 was added to one culture at T0 and one when the culture reached an equivalent OD_600_ (~0.1) as when the cells were grown to T3 at 37 ^o^C (Fig. S3a). Additionally, we slowed down the growth rate by growing cells in M63-lactose minimal media (37 ^o^C) to an OD_600_ of 0.1 (Fig. S3b). In both conditions of slower growth rates, cells were able to grow better when A22 was added at a later time point than at T0. These results suggest that differences in growth rate are not the likely cause for A22 resistance at T3.

**Fig. 2:**
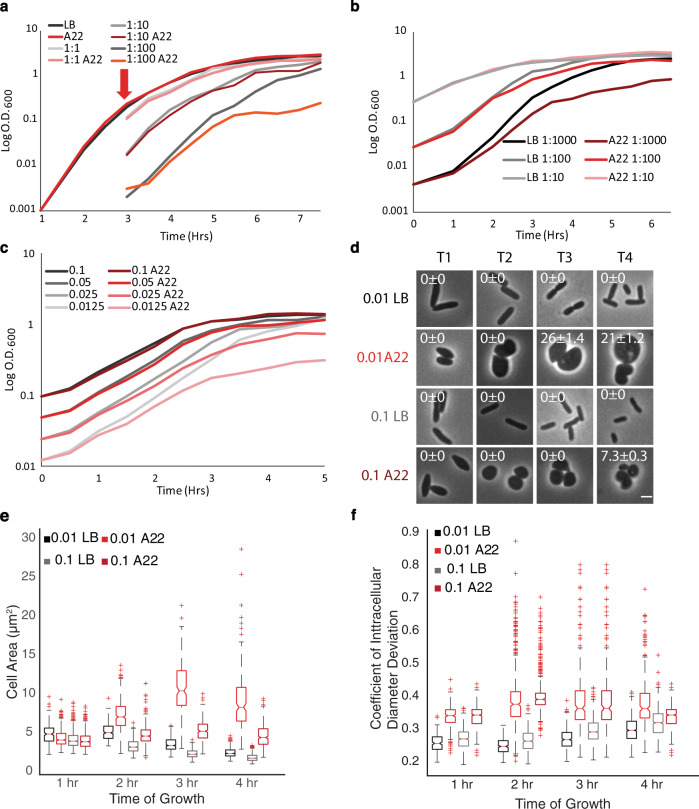
Growth resistance is dependent on cell density. **a** Representative log-scale growth curve of WT cells grown at 37 °C treated with A22. Cells were grown in LB without A22 for three hours (arrow) and then either allowed to continue to grow, spiked with A22, or back diluted into pre-warmed media with and without A22 (10 μg/ml) at the indicated dilution factors. **b** Representative log-scale growth curve of WT cells grown at 37 °C with different inoculation dilutions. Cells were grown with and without A22 (10 μg/ml) in LB at the indicated initial inoculation ratio from overnight cells. **c** Representative log-scale growth curve of wild-type cells with different starting ODs. Overnight cells were washed and resuspended into new MOPS media and inoculated at the indicated OD_600_ with and without A22 (10 μg/ml). Growth rates for each starting OD can be found in Table S3. **d** Representative phase-contrast images of WT cells grown at 37 °C from OD_600_ ~0.01 or 0.1 with and without A22 (10 μg/ml). Cells were imaged every hour for 4 h. White arrow indicates cell vesicles forming in advance of lysis and numbers in the upper left represent the percentage of cells displaying vesicles and the Poisson error to one standard deviation. The growth curve can be found in Fig. S3d. The scale bar is equal to 2 µm. **e**, **f** Quantification of cells imaged in **d**. Red pluses are outliers, the red line is the median, the bottom and top edges of the box represent 25th and 75th percentiles, respectively, and notches are 95% CI. Data were pooled from three independent experiments. **e** Cell area from cell imaging in **d**. **f** Coefficient of intracellular diameter deviation from cell imaging in **d**. **e**, **f** Number of cells can be found in Table S4.

Next, we investigated whether the growth phase was responsible for the WT-like growth in A22, as T0 cells are in the lag phase and show reduced growth while T3 cells are in the exponential phase and grow at a rate as if they were in LB only medium. To mimic the growth conditions of cells at T3, we subcultured cells grown to T3 into pre-warmed media with A22 (LB_A22_) and without (LB) A22 (Fig. 2a and S4). At a 1:100 dilution of T3 cells, A22 causes a significant reduction in growth rate, leading us to conclude that the exponential phase alone is not the reason for continued growth after the A22 addition. We then determined if different subculture dilutions would show similar results. Interestingly, 1:1 and 1:10 dilutions grow the same whether or not A22 is present (Fig. 2a and S4). A major difference between the different dilutions is the number of cells present; therefore, we hypothesized that cell density was responsible for the observed growth resistance. However, as smaller dilutions allow for less mass doublings before cells reach the stationary phase, it is possible that due to the slow action of A22 the number of possible mass doublings results in the differences in growth.

To test this, we repeated the back dilution experiment with cells grown to an OD_600_ ~0.1. These cells were back diluted 1:1 into pre-warmed media with and without A22 and allowed to grow again to an OD_600_ ~0.1. This was repeated for a total of five “generations” (Fig. S3c). There is no observed growth defect due to A22 over the course of these “generations” despite allowing cells to have more time in exponential phase and therefore more mass doublings. This further suggests that the slow action of A22 is not responsible for the observed growth differences.

To test the effect of cell density on the ability of the cells to continue to grow in A22, we grew cells in A22 in two ways: (1) using different inoculation ratios from an overnight culture and (2) using different starting ODs to begin the growth curves, both with A22 the entire time. Our previous growth curves began with a 1:1000 dilution of an overnight culture. We decreased this dilution factor by an order of 10 or 100 and saw a decrease in the difference of growth rates between cells grown in A22 medium compared to medium alone as the dilution factor increased, supporting our hypothesis that cell density is important for growth in A22 (Fig. 2b and S5). Cultures starting with specific ODs also show a density-dependent growth phenotype, further supporting the role of cell density in A22 growth resistance. To avoid any media effects, an overnight culture was washed in fresh media before cells were inoculated in media with or without A22 at an OD_600_ of 0.1 (~the same as T3 cells), 0.05, 0.025, and 0.0125. The higher starting density cells show no growth difference with or without A22 (Fig. 2c, S6, and Table 1). However, inoculation of cells at lower O.D.s results in a change in growth rate when A22 is added. (Fig. 2c and S6). To quantify the growth rate changes at the different starting ODs we measured the exponential growth rate of cells from each dilution and compared the growth in media with and without A22 and averaged across repetitions (Table 1 and Table S3). These results show that the difference in growth rates between media with and without A22 is only statistically significant when the cell density is low; at higher starting ODs there is no difference in growth between media with and without A22. These data further refute the idea that the growth phase is important for the growing resistance, as all cells are starting in the lag phase, independent of starting inoculum density, and support the hypothesis that the ability of *E. coli* to grow as round cells with disrupted MreB is dependent on cell density. In addition, all starting OD_600_ conditions grew linearly for similar amounts of time and only the lower cell densities showed growth defects, implying there was an equal amount of mass doubling for A22 to have an effect.

**Table 1:** Increased cell density allows cells to grow in A22.

Starting OD_600_	A22 growth rate/MOPS growth rate	SD	*P* value vs 0.1	*P* value vs .05
0.1	0.999	0.0345	Na	0.851
0.05	0.956	0.0391	0.851	Na
0.025	0.800	0.1200	**0.025**	0.078
0.0125	0.691	0.0171	**0.002**	**0.005**

*SD* standard deviation.

Growth rate ratio averages of cells grown in A22 (10 μg/ml) vs MOPS from triplicate experiments. Students *t*-test to compare starting OD_600_ 0.1 group vs all other starting OD_600_ groups and starting OD_600_ 0.05 group vs all other starting OD_600_ groups. Bold indicates statistically significant differences.

As modulating cell density phenocopies the A22 growth pattern that was initially observed for cells treated at different time points (Figs. 1,  2c), we tested if increasing cell density results in the same physiological changes to A22 treatment: vesicle formation and cell size. Cells were grown from a starting OD_600_ of ~0.1 or 0.01 in LB or LB_A22_ and imaged every hour for 4 h (Figs. 1e, 2d). Unlike our previous experiment, this setup also accounts for differences in the length of A22 exposure as all cultures received A22 at the beginning of the assay. Both treated cells become round at the same rate and to the same degree when treated with A22, supporting the idea that the degree and speed of MreB depolymerization is unchanged (Fig. 2f). Interestingly, 0.1 cells did not become as large as their 0.01 counterparts (Fig. 2e), similar to what was seen for T1 and T3 cells (Fig. 1d). Although both inoculation densities result in vesicle formation, the low-density cells form vesicles earlier and at a higher frequency than the high-density cells (Fig. 2d). These data support the hypothesis that cells at different densities have different physiological responses to A22. We will refer to this phenomenon as density-dependent growth resistance (DDGR).

It is possible that cells might serve as a sponge or reservoir for A22 by physically adsorbing the A22 to the membrane or collecting it in their cytoplasm thereby diluting out the drug. To test this idea, we artificially increased the starting OD of culture with intact heat-killed cells (inset Fig. S3e). Heat-killed cells were added to fresh media at an OD of 0.4, a concentration >4X what is needed to observe A22 growth resistance. Cells grown overnight were subcultured 1:1000 into this culture with and without A22. The presence of heat-killed cells did not protect the subcultured cells from the A22, as there is an obvious separation of growth between the LB and LB_A22_ media (Fig. S3e). This suggests that some component of live cells is responsible for DDGR. In addition, DDGR does not go away even with a 5X increase in the A22 concentration added at T3 (Fig. S7a) supporting the idea that physiological differences exist between cells at different densities.

### Changes in the media are not responsible for A22 growth resistance

**Fig. 3:**
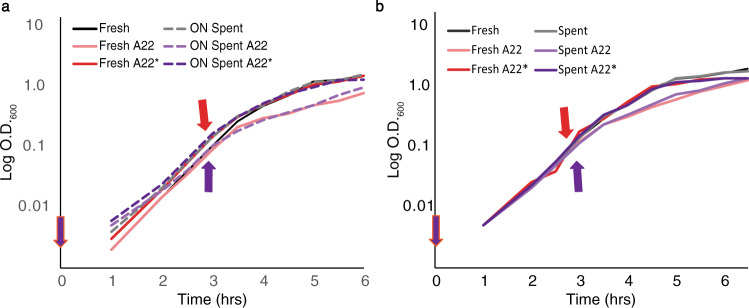
Changes in media do not cause growth resistance. Arrows are color-coded to the lines representing spike (*) conditions. **a** Representative growth curve of WT cells grown at 37 °C with 10% spent media from an overnight culture. Cells were grown in LB, A22 (10 μg/ml), and A22 added at the indicated time where OD_600_ ~0.1 (*). **b** Representative growth curve of WT cells grown at 37 °C with 100% spent media from a culture grown to an OD_600_ 0.1. Cells were grown in LB, A22 (10 μg/ml), and A22 added at the indicated time where OD_600_ ~0.1 (*).

Our results suggest that cell density is important for A22 growth resistance. Because we washed the overnight cells before use, we did not think quorum sensing (QS) or media changes were involved. However, to specifically test a role for QS, we grew cells in 10% spent media from overnight cultures with and without the addition of A22^30^. The addition of 10% spent media did not confer resistance to cells grown in A22 (Figs. 3a and S8c, d), suggesting that QS is not involved in A22 growth resistance.

As cells grow, they induce other changes in the medium, such as a decrease in pH, an increase in waste products, and the removal of nutrients^31–35^. To test if any of these changes were occurring and responsible for the increased growth in A22, we grew cells with A22 in 100% partially spent media from cells grown to an OD_600_ ~0.1 (T3). As cells at T3 exhibit DDGR, if the resistance is due to the media conditions at this time point, freshly inoculated cells should also exhibit A22 growth resistance. We found no difference in growth in cells treated with A22 in the 100% partially spent media vs the fresh media (Fig. 3b and S8a, b). These results suggest that, while living cells are required for DDGR, cells are not making a diffusible product nor are they changing the media to induce resistance.

### Cell density provides resistance to PBP2 disruption

**Fig. 4:**
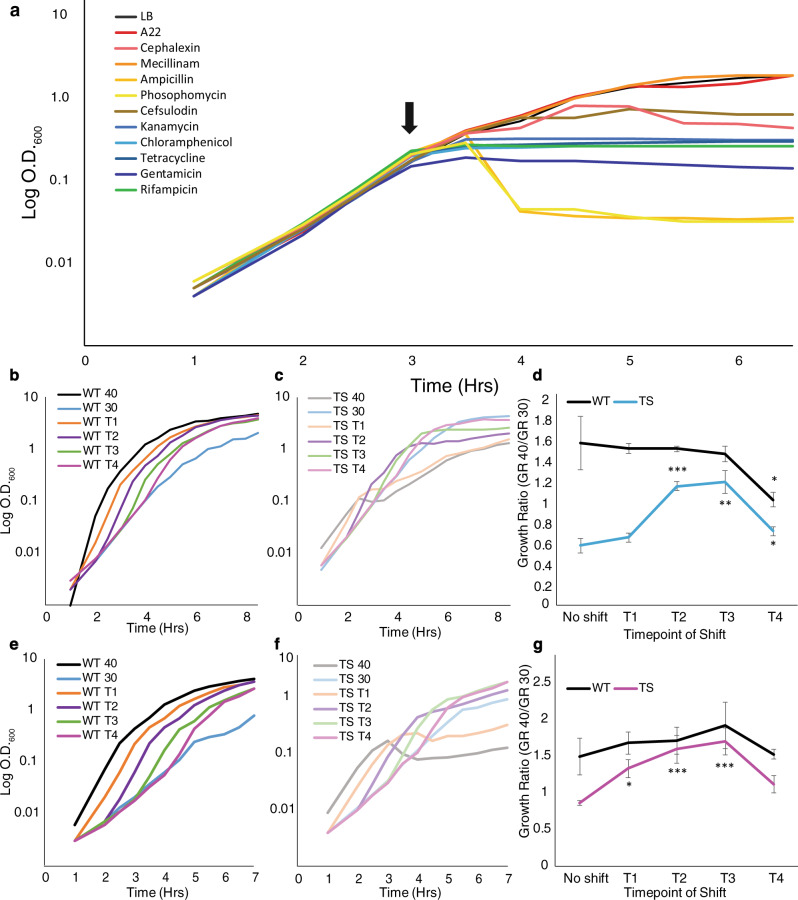
PBP2 disruption also demonstrates growth resistance. **a** Representative growth curve of WT cells grown at 37 °C treated with a variety of antibiotics at the indicated time (arrow). A22 (10 μg/ml), cephalexin (10 μg/ml), mecillinam (3 μg/ml), ampicillin (100 μg/ml), phosphomycin (10 μg/ml), cefsulodin (30 μg/ml), kanamycin (30 μg/ml), chloramphenicol (35 μg/ml), tetracycline (10 μg/ml), gentamycin (45 μg/ml), and rifampicin (500 μg/ml). **b**–**g** Cells were grown in LB supplemented with glycerol at 40 °C, 30 °C, and shifted from 30 to 40 °C every hour for 4 h. **b** Representative growth curve of WT. **c** Representative growth curve of PBP2ts cells. **d** Pooled average growth rate ratios (growth rate at 40 ^o^C/growth rate at 30 ^o^C from WT and PBP2ts. Cells were grown at 30 ^o^C and transferred to 40 ^o^C every hour for 4 h. **e** Representative growth curve of WT cells. **f** Representative growth curve of RodAts cells. **g** Pooled average growth rate ratios (growth rate at 40 ^o^C/growth rate at 30 ^o^C from WT and RodAts. Cells were grown at 30 ^o^C and transferred to 40 ^o^C every hour for 4 h. **d**, **g** Error bars are standard deviations from three biological replicates. Significance was determined using the student’s *t*-test of T1–T4 growth ratios vs the no-shift control for each strain. For WT there was no significant difference. For RodAts cells: T1 − *p* = 0.05, T2 − *p* = 0.0012, T3 − *p* = 0.0002.

A22 is not a clinically used antibiotic; therefore, we wanted to determine if DDGR is common among other antibiotics, including those used clinically. We tested *E. coli* cells grown to T3, to equalize time of drug exposure, and added inhibitory doses of antibiotics targeting cell wall synthesis (A22, mecillinam, cephalexin, cefsulodin, phosphomycin, and ampicillin), translation (kanamycin, chloramphenicol, gentamicin, and tetracycline), and transcription (rifampicin) (Figs. 4a and S9). The translation- and transcription- targeting antibiotics arrest growth within 30 min of the addition of the antibiotic, while the cell wall-targeting antibiotics cause more variations in response. Phosphomycin and ampicillin both cause active cell lysis within an hour after addition while cells treated with cefsulodin and cephalexin grow for an hour before growth is inhibited; however, mecillinam (mec), which specifically targets PBP2, is the only tested antibiotic that phenocopies the A22 DDGR phenotype. As MreB (target of A22) and PBP2 (target of mec) interact with each other, it is logical that they both show this phenomenon when targeted by their respective antibiotics^14,36^.

Similar to A22, mec also causes treated cells to become swollen and round (Fig. S10), confirming that mec is still active when added at higher cell densities. To confirm that the concentration of mec used is high enough to kill cells when added at lower ODs, we performed a growth curve analysis with mec added at T0 and T3. The concentration of mec used is high enough to almost abolish all growth when added at T0 but shows almost no effect when added at T3 (Fig. S11b).

It has been reported that cephalexin causes a similar density-dependent resistance as we have observed for A22 and mec^37,38^. Because we did not observe cephalexin to have DDGR we replicated the experiment of Chung et al. which grew cells at different starting dilutions with cephalexin^37^. It was reported that cephalexin has no effect when a 1:100 starting dilution is used compared to a 1:1000 dilution. Repeating this experiment, we do not see the same growth in cephalexin at an initial inoculation of 1:100. However, if we use a 1:10 starting dilution we are able to recapitulate their results (Fig. S11a). In this respect, we believe that resistance to cephalexin at a 1:10 inoculation ratio is likely a result of limited mass doublings as these cells barely grow and is a different phenomenon than DDGR reported here.

To determine if the DDGR seen with mec treatment was due to mec itself or the inhibition of PBP2, we used a temperature-sensitive mutant of PBP2 (PBP2ts)^39^. At temperatures >40 ^o^C, the PBP2ts mutant grows slowly, and the cells are round, while at the permissive temperature (30 ^o^C) the cells grow as rods. To test if the growth defect associated with the loss of PBP2 is density-dependent, we started both WT and PBP2ts cultures growing at 30 ^o^C and shifted them to 40 ^o^C every hour for 4 h. If there is a DDGR phenotype, the change in growth rate after the shift to the higher temperature should be greatest when cultures are shifted early, before the proper density is reached. As a control, we grew the WT and PBP2ts cells at both temperatures the entire time (Figs. 4b, c and S12). WT cells are able to grow well at both temperatures, while the PBP2ts strain has reduced growth at 40 ^o^C. In order to determine how growth was affected by the shift in temperature, we measured the ratio of growth rates of each strain before and after the temperature shift from each repetition (Fig. 4d). For cultures shifted at T1 and T2, there were not enough data points to calculate a growth rate during the 30 ^o^C growth period; therefore, we used the growth rate of the control sample. In support of our density-dependent hypothesis, at all time points, WT cells show an increased growth rate when shifted to 40 ^o^C (above 1 Fig. 4d), while the PBP2ts mutant grows worse after the shift for the first 2 h (below 1 Fig. 4d). After 3 h of growth at the permissive temperature, the change in growth rate caused by the temperature shift was minimal, likely due to the cells being at a high enough density for DDGR (Fig. 4c, d). This result, in conjunction with the mec and A22 experiments, further supports the hypothesis that the elongasome can be disrupted at higher cell densities without deleterious effects on cell growth.

With two essential components of the elongasome displaying DDGR, we sought to test RodA, the essential transglycosylase partner of PBP2, for DDGR^6,28^. While there are no antibiotic compounds we are aware of that can inhibit RodA, there is a temperature-sensitive mutant^40^. As such, we performed the same temperature shift experiment with RodAts cells as we had with PBP2ts cells. WT cells displayed the same pattern of growth in shifts as they had previously (Figs. 4e and S13). While RodAts cells grew more poorly overall, they still display better growth at 40 °C after later shifts even when averaged across all repetitions (Fig. 4g). Interestingly, the T1 shift results in cells stopping growth and lysing before growth is continued (Figs. 4f and S13). These results support the model that the specific inhibition of the elongation machinery can be overcome in a density-dependent manner.

### PBP1B compensates for disruption of the elongasome

In addition to the MreB-PBP2-based elongasome complex, there are other cell wall synthesis enzymes, including PBP1A and PBP1B. Unlike PBP2 which only has transglycosylase activity, PBP1A and PBP1B have both transglycosylase and transpeptidase activity^41–43^. While PBP1A and PBP1B can compensate for each other, they are proposed to have different functions, with PBP1B being in the divisome and PBP1A working semi-autonomously of MreB during elongation^12,13^. We have recently shown that a mutation in malate dehydrogenase, *mdh*, is more tolerant to A22 and mec and that this increased resistance is possibly due to the increased activity of PBP1B^17^. Therefore, we sought to determine if either PBP1A or PBP1B are needed for continued growth after A22 or mec treatment at higher cell densities.

**Fig. 5:**
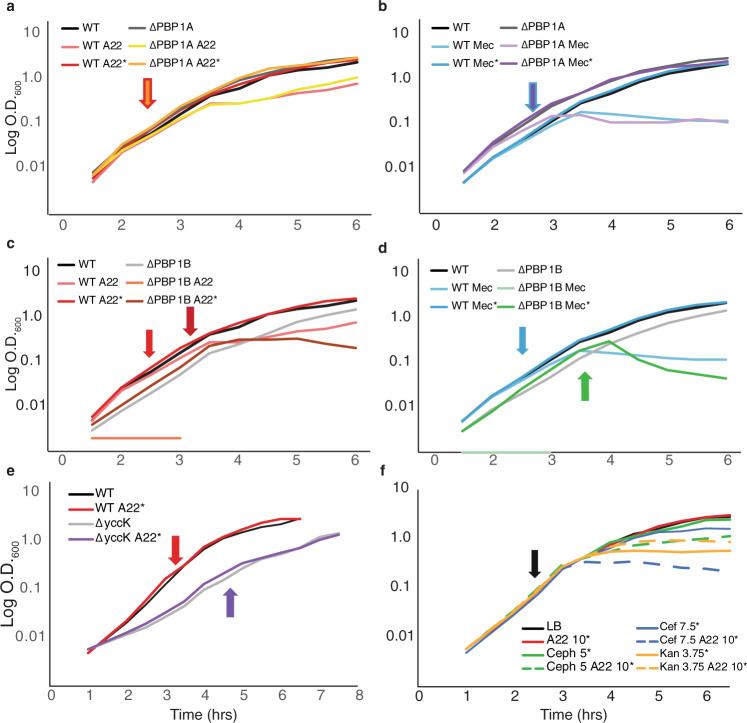
PBP1B is responsible for density-dependent growth resistance. Representative growth curves cells grown at 37 °C in LB. **a** WT and ΔPBP1A cells were grown in A22, A22 (10 μg/ml), and A22 added at the indicated times where OD_600_ ~0.1 (*). Arrow coloration corresponds to which strains were spiked at that time. **b** WT, ΔPBP1A cells were grown in mecillinam, mecillinam (3 μg/ml), and mecillinam added at the indicated time per strain where OD_600_~0.1 (*). **c** WT and ΔPBP1B cells were grown in A22, A22 (10 μg/ml), and A22 added at the indicated times where OD_600_ ~0.1 (*). Arrow coloration corresponds to which strains were spiked at that time. **d** WT and ΔPBP1B cells were grown in mecillinam, mecillinam (3 μg/ml), and mecillinam added at the indicated time per strain where OD_600_~0.1 (*). **e** WT and ΔtusE cells in LB and A22 (10 μg/ml) were added at the indicated time per strain where OD_600_~0.1 (*). **f** Representative growth curve of WT cells grown at 37 °C in LB without antibiotics and the indicated antibiotics at the concentrations (μg/ml) added at the indicated time where OD_600_ ~0.1 (arrow and *).

We tested ΔPBP1A and ΔPBP1B deletions for their ability to continue to grow after spikes of A22 or mec at OD_600_ ~0.1 compared with the addition of A22 at T0. When spiked with either A22 or mec, ΔPBP1A cells continue growing at the same rate as the LB only control. However, the ΔPBP1B cells stop growing within an hour and eventually lyse when spiked with A22 or mec (Figs. 5a–d and S14), supporting previous reports^44,45^. These data suggest that PBP1B is needed for DDGR of both A22 and mec treatments.

We noticed that the PBP1B deletion cells are more sensitive to both A22 and mec compared to WT when added at T0 (Figs. 5a–d and S14). To confirm that the lack of DDGR to mec and A22 in the PBP1B mutant is not a result of its increased sensitivity to these drugs, we checked if other A22-sensitive mutants maintain DDGR. To our knowledge, there are no known mutations outside of MreB that increase sensitivity to A22; therefore, we performed an A22 sensitivity screen on the Keio collection in order to find such mutants^46^.

From this screen, we have confirmed two mutants with increased sensitivity to A22: *envC* and *yccK* (*tusE*) (Fig. S15a). EnvC is an activator of cell wall hydrolases needed to separate daughter cells after division^19^. Due to the suspected role of PBP1B in survival after A22 or mec treatment and PBP1B’s proposed role in cell division, we decided to focus on the *tusE* deletion as our control-sensitive strain. TusE is a sulfur transferase involved in the modification of tRNA and is, therefore, a good candidate to use as a control for these experiments^47^. We tested Δ*tusE* cells for their ability to survive a spike of A22 or mec at an OD_600_ ~0.1 and found no reduction in growth despite the heightened sensitivity of these cells when the drugs are added at T0 (Figs. 5e and S15b, S16, S17). This indicates that the inhibition of growth and lysis after the addition of A22 or mec at later time points is due to the lack of PBP1B and is not a universal feature of strains more sensitive to A22 or mec.

As an A-class PBP, PBP1B has two enzymatically active domains; the transglycosylase domain (TG) and the transpeptidase domain (TP)^48^. We wanted to determine if these two domains have differential roles in PBP1B’s essentiality for round cell growth and DDGR. We grew strains with a defective TG domain, defective TP domain, or both domains defective and added A22 at an OD ~0.1 (Figs. S15c and S18). All versions of a defective PBP1B do not display DDGR indicating that a completely functional PBP1B is necessary for DDGR.

Previous data have shown that overexpression of cell division proteins suppresses the growth defects of a *mreB* deletion^28,49^. Our current work shows a role for PBP1B in the survival of cells treated with A22, further suggesting that the divisome is important during growth and not just division (Fig. 5a–d). To further test the importance of the divisome during A22 treatment, we treated cells with combinations of A22 and antibiotics that inhibit a divisome component: cephalexin (FtsI), cefsulodin (PBP1A/B), or a control: kanamycin (30 S ribosomal protein). We used a dose of each antibiotic that is closer to the minimum inhibitory concentration (MIC) and lower than what was used in Fig. 4 (cephalexin, 5 µg/ml; cefsulodin, 7.5 µg/ml; and kanamycin, 3.12 µg/ml), to more easily determine changes in growth due to A22. Each drug was added when cells reached an OD_600_ ~0.1 alone or in combination with A22. When cells are treated with this lower concentration of cephalexin there is minimal growth inhibition. However, when A22 is added at the same time cell growth is dramatically reduced. When cefsulodin is used alone, cells stop growing after ~2 h, but when A22 is added with cefsulodin, cells stop growing within 1 h and reach a lower OD. The kanamycin control-treated cells stop growing after the addition of the drug, but when kanamycin is combined with A22, cells do not display an increase in sensitivity compared to the kanamycin-only treated cells (Fig. 5f and S19). These data show that inhibiting divisome components, but not other cellular targets, blocks the A22 density-dependent growth resistance phenotype, supporting the idea that the divisome is needed for growth after disruption of MreB and reinforcing a role for PBP1B in DDGR.

### DDGR is regulated posttranscriptionally

To determine if DDGR is caused by transcriptional changes in cells at a higher density we examined the literature for RNAseq data comparing cells from T0/1 to T3. With a focus on genes involved in cell wall synthesis (Fig. S20a, c), coordination (Fig. S20b), and recycling (Fig. S20d) we used the data from Smith et al. comparing expression changes from stationary phase *E. coli* (BW25113) to cells at T0–7^50^. The majority of the *fts* genes were excluded from this analysis as gene expression was similar to *ftsW* and *ftsZ*. The goal was to find genes whose regulation was different at T3 than either T0 or T1 in order to account for the transcriptome shift from pre-DDGR to post-DDGR conditions (Fig. S20a–d). Of the 39 genes, we looked at only four show an expression pattern that would be consistent with what we expect from genes involved in DDGR. These four genes are *yfeW* (PBP4B), *murR*, *murP*, and *murQ*. We performed growth curves on strains with deletions in these genes when A22 was added at an OD_600_ ~0.1. Deletions of each of these genes had no effect on these cells’ ability to perform DDGR (Fig. S20e and S21). This indicates that the mechanism(s) behind DDGR either occurs posttranslationally and/or in systems not directly related to the cell wall.

### A22 spike resistance is common in MreB rod species

**Fig. 6:**
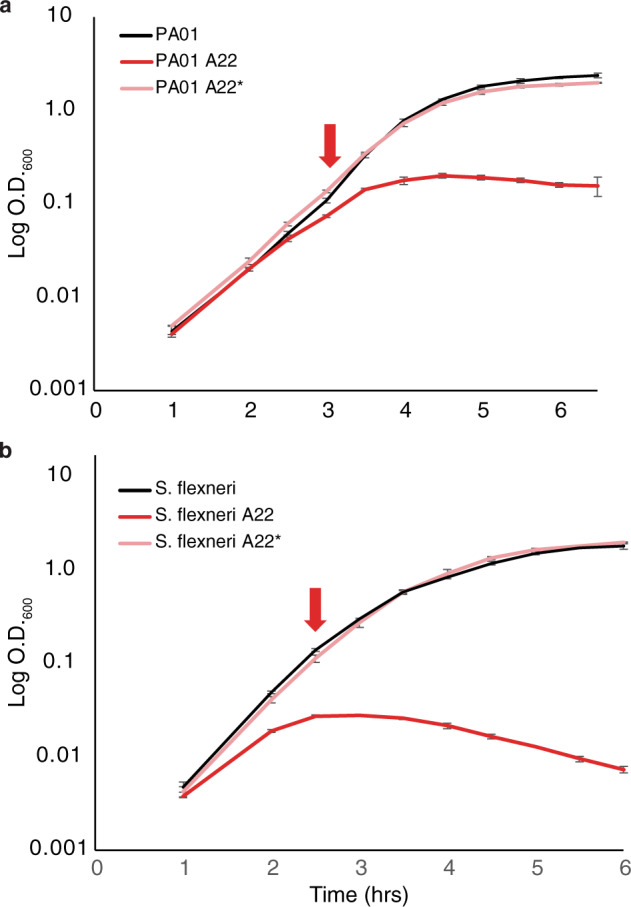
Other Gram-negative rod species display DDGR. **a** Growth curve of Pseudomonas aeruginosa PA01 grown at 37 °C in LB, LB_A22_ (20 μg/ml), and LB with A22 added at the indicated time point where OD_600_ ~0.1 (*). **b** Growth curve of *Shigella flexneri* (ATCC 12022) grown at 37 °C in LB, LB_A22_ (10 μg/ml), and LB with A22 added at the indicated time point where OD_600_ ~0.1 (*). Error bars are standard deviation from three biological replicates.

MreB is highly conserved in many rod-shaped organisms, including multiple human pathogens making a potential target for antibiotic development. To assess if DDGR is a common phenomenon among MreB-containing bacteria we tested two human pathogens, *Shigella flexneri* and *Pseudomonas aeruginosa*. We performed growth curves using both *P. aeruginosa* PA01 and *S. flexneri* with A22 added either at T0 or an OD_600_ ~0.1 (Fig. 6). Like *E. coli*, both species continued to grow similar in LB and LB_A22_ only after the addition of A22 at OD_600_ ~0.1. Both species grew worse when A22 was added at T0, further supporting the role of cell density in resistance to A22. We show that DDGR to the loss of MreB is found across three distinct genera indicating a common mechanism for growth during elongasome inhibition.

## Discussion

MreB is necessary for proper cell shape in many rod-shaped bacteria. MreB polymers are critical for the proper insertion of peptidoglycan in rod-shaped cells, as they are thought to organize the localization of the cell wall synthesis enzymes^1,3,12^. We, therefore, hypothesized that rapidly growing cells, such as those in the exponential phase, would be more sensitive to disruption of MreB than slow-growing cells, such as those in the stationary phase. In fact, the transcriptional repressor BolA reduces MreB levels during the stationary phase^20,22^. To test this idea, we added the MreB depolymerizing drug A22 during the beginning and end of the exponential phase expecting to see less of an effect as cells approached the stationary phase. Surprisingly, we found no growth defect when A22 was added in the early exponential phase (Fig. 1a). Furthermore, the growth resistance seen in response to A22 treatment is a result of cell density. Density-dependent growth resistance (DDGR) to the loss of the elongasome is only possible when PBP1B is present.

Cells treated with A22 at the time of inoculation (T0) show a growth defect, but cells treated with A22 2–3 h after inoculation continue to grow at the same rate as cells in LB only medium (Fig. 1a). Cells treated at T1 become much larger and stay that size compared to those treated at T3 which continue to grow in size after the A22 treatment. Additionally, T1 cells develop intracellular vesicles, indicating the beginning of cell lysis (Fig. 1b), while T3 cells do not^27^. It is unclear why the timing of A22 treatment causes such a drastic change in cell area and vesicles formation, but this suggests differences in the physiological state of the cells and may lead to insights into the cause of survival differences (Figs. 1b, c and  2d, e). Similar vesicle-like structures have been observed when the elongasome is inhibited and is most likely a general consequence of growth as a large sphere^28,51^. Depletion of any of the *mre* genes results in these internal structures, which has been shown to be true vesicles^28^. It has been proposed that spherical cells produce membrane at the same rate as rod cells, which due to the increase in volume-surface ratio in spherical cells would lead to excess membrane^52^. The formation of internal vesicles could be a mechanism for cells to remove this excess membrane. Interestingly, we see less vesicle formation in cells able to perform DDGR (Figs. 1b, 2c), suggesting that loss of rod shape is not sufficient to cause vesicle formation, but that cell size is also important as cells in a DDGR state have a smaller volume and less commonly contain vesicles, even though they become spherical. The mechanism linking cell size to cell density is not clear.

We have shown that the growth phase is not responsible for DDGR as the dilution of exponential phase cells results in sensitivity and lag phase cells can be resistant when inoculated at different cell densities. (Fig. 2). Together these results suggest a density-dependent mechanism for growth resistance. Because it is possible that higher amounts of cells lead to dilution of the antibiotics, we added 5X more A22 and 33X more mecillinam (Fig. S7) without losing DDGR, suggesting that the increased number of cells are not diluting out the antibiotic. Finally, temperature-sensitive mutants of both PBP2 and RodA show DDGR without the use of drugs (Fig. 4b–g).

Cell-cell interactions might play a role at higher cell densities but given that all experiments took place under conditions of shaking liquid media, any cell-cell contact is likely to be brief. It is possible that the likelihood of cell-contact increases in a density-dependent manner even in shaking culture; however, other cell contact-dependent phenomena such as killing via type VI secretion or conjugation occur on solid media but not in liquid^53–55^.

It is suggested via BolA’s activity that cells in stationary phase and therefore at T0 should have less MreB^20,22^. It is possible that the lower amount of MreB potentially increases the effectiveness of A22, as there would be more A22 per each MreB polymer per cell; however, we don’t believe this explains DDGR as multiple experiments have shown a density dependency of cells in different growth phases. In testing the role of exponential phase in DDGR we saw that log-phase cells (high MreB concentration/cell) and stationary phase cells (low MreB concentration/cell) can be desensitized to A22 (Fig. 2a–c) by simply changing the cell density: indicating DDGR is not related to the ratio of drug to target protein.

Nonbiological differences cells encounter between T0 and T3 are changes in the media. When cells grow in LB, they exhaust certain nutrients in a stepwise fashion. When one nutrient is exhausted, they alter their metabolism and that shift in metabolism can alter physiology and potentially A22/mec resistance^31,56^. Additionally, as bacteria metabolize amino acids, they lower the pH of the media, and lower pH has been linked to increasing MICs^56^. That cells were grown in spent media from OD_600_ ~0.1 cells still display sensitivity to A22 at T0 and DDGR at T3 (Fig. 2c) further supports the idea that changes to the media are not the cause of DDGR. To further our conclusions about pH we have also performed experiments in MOPS (Fig. 2c) and M63 minimal media (Fig. S3) which do not show as dramatic pH changes as seen in LB. Therefore, we can conclude that nutrient exhaustion and pH changes are not the cause of DDGR.

A common cell density-dependent phenomenon is quorum sensing (QS). QS has been linked to antibiotic resistance via efflux pump activity, and biofilm formation^30,34,57^. We have shown that QS is not involved in density-dependent growth resistance (DDGR). When 10% overnight spent media was used, we saw no change in growth between A22 and LB only media at T0 (Fig. 3b). Additionally, when treated with A22 at T3 the cells growing in 10% spent media displayed the same growth rate as those in fresh LB. These data suggest that DDGR is not due to QS.

To determine if DDGR is a common feature of other antibiotics, we tested ten additional antibiotics with a variety of cellular targets (Fig. 4a). DDGR was only observed in A22 and mec, which targets PBP2 (Fig. 4a and S11b). This is interesting because while A22 and mec do not share the same protein target, MreB, the target of A22 directly interacts with PBP2, the target of mec, in the elongasome complex^3,13,15^. In addition, a similar phenotype has been reported for cephalexin treatment based on an observation that a 1:100 dilution of cells are resistant to cephalexin while a 1:1000 dilution of cells is sensitive^37^. While attempting to replicate this experiment unsuccessfully, we noticed that the reported starting OD of the 1:100 dilution more closely resembled that of a 1:10 (Fig. S11a). To more closely replicate the published results, we repeated the experiment with a 1:10 dilution and saw that cells become resistant to cephalexin although there are little mass doublings. This suggests that the action of cephalexin is very sensitive to the number of mass doublings cells undergo resulting in a separate phenomenon from the DDGR reported here.

To confirm that the DDGR observed from mecillinam treatment is due to specific inhibition of PBP2 we shifted a temperature-sensitive mutant of PBP2 (PBP2ts) to the nonpermissive temperature (40 ^o^C) at different densities (Fig. 4c). Similar to A22 and mec addition, we found a density dependency on growth, as cells shifted at higher densities continue to grow better after the temperature shift. These data suggest that the elongasome is less essential at specific cell densities while overall peptidoglycan synthesis remains essential. Another essential elongasome component is RodA, the transglycosylase partner of PBP2^5,6^. Similar to PBP2, a RodAts mutant displays better growth in restrictive temperatures at higher cell densities.

When mec was first developed in 1972, most experiments focused on efficacy and method of action^8,58,59^. Mec’s narrow target range allowed for precise exploration of the elongasome leading to the discovery of the Mre family of proteins^18^. Some researchers observed differences in cell survival to mecillinam based on density; however, to the best of our knowledge, no mechanism has ever been put forth^8,60^. In the section below, we outline a possible mechanism for cell survival after A22 or mecillinam treatment.

The addition of A22 and mec at later time points does not reduce the growth rate of cells but does result in cells becoming round, indicating A22 and mecillinam are still able to inhibit their targets. These round cells continue to grow, both at the population level (OD) and single-cell level (cell area) but do not lyse like cells treated with A22 at earlier time points (Fig. 1), suggesting a possible role for another peptidoglycan synthase. Inhibition of either the TP or TG domain of PBP1B is sufficient to block DDGR, suggesting that a fully functional PBP1B is needed to compensate for the loss of the elongasome in a density-dependent manner, and further refuting the idea that these specific antibiotics are being diluted out by increased cell numbers (Fig. 5a–d).

PBP1A and PBP1B are aPBPs capable of performing both transglycosylase and transpeptidase activities and have been shown to work semi-autonomously from the MreB based elongasome^5^. It has been suggested that PBP1B is involved in the division while PBP1A is involved in elongation^5,48^. Why and how PBP1B and the divisome alter activity in a cell density-dependent manner is unclear. While PBP1A and PBP1B can compensate for each other, PBP1B does have distinct functionality from PBP1A. PBP1B’s transglycosylase domain is essential for de novo peptidoglycan synthesis, while PBP1A is unable to perform de novo peptidoglycan synthesis^44,45,48^. However, if either the transglycosylase domain or the transpeptidase domain are inhibited DDGR cannot occur. Interestingly, While PBP1B is mostly known for its role in the divisome there have been links to a role in the elongasome via A22 and mec sensitivity^17,61^. Additionally, our work shows a completely functional PBP1B is necessary to overcome the loss of PBP2 and MreB in a density-dependent manner as both ΔPBP1B cells or cells inhibited in either the TP or TG domain lyse when treated with mec or A22 (Fig. 5a–d and Fig. S15c). It is possible that PBP1B is playing some role in linking elongasome and divisome activity.

In further attempts at elucidating a mechanism for DDGR we investigated published RNAseq data to understand the transcriptional profile of cells at low (T0) or high (T3) density. Examination of the changes in transcript levels of many cell wall-related genes found four genes displayed a predicted change in transcription. Strains deleted for these genes were still able to undergo DDGR, suggesting that a mechanism for either the round cell growth or the non-QS density sensing lies outside of genes typically associated with the cell wall or occurs posttranslationally (Fig. S20). Our current model is that PBP1B activity is modulated posttranslationally in a cell density-dependent manner so that at higher cell densities PBP1B is more active and better able to compensate for the loss of elongasome activity.

The ability of bacteria to evade killing from antibiotics is an important clinical feature. In addition to being resistant to the effects of an antibiotic, which results in a higher MIC and is caused by heritable mutations, cells can avoid being killed through biofilm formation, antibiotic tolerance, and the formation of persister cells. Biofilms act as a physical barrier to block the diffusion of antibiotics keeping them away from the cell^62^. Tolerance and persistence both temporarily avoid cell death either by modifying growth rate or forming a metabolically inactive subpopulation^63,64^. All three of these avoidance mechanisms can lead to mutations that eventually result in the accumulation of resistance. Here, we show that DDGR is another method for bacteria to evade the effects of antibiotics.

The cell wall has always been an attractive antibiotic target due to its universality in bacteria and the lack of a peptidoglycan cell wall in eukaryotic cells. Most Gram-negative rod-shaped pathogens utilize a Mre/Rod family-based cell wall synthesis system^65^ and therefore might display DDGR. The rod-shaped pathogens, *Pseudomonas aeruginosa* and *Shigella flexneri*, display DDGR in response to A22 (Fig. 6).

Growth in the presence of antibiotics due to DDGR can potentially allow cells to develop true resistance to antibiotics. Antibiotic cocktails have been long used as a method of treatment in antibiotic-resistant infections^66,67^. One complication of cocktails is that a higher number of drugs included in the cocktail increases the likelihood of complex interactions, thereby making them less effective^67,68^. As a best practice, drug cocktails should be kept to the minimum number of antibiotics needed to avoid these interactions and to minimize creating a super-resistant infection^68^. Simultaneous disruption of the elongasome and divisome, as seen in the cefsulodin-A22 or the cephalexin-A22 treated cells (Fig. 5f), is a way of overcoming DDGR. This is supported by both demonstrating peptidoglycan synthesis to have a division of labor between the divisome and elongasome and each having their own essential enzymes and proteins^5,16,37,45^ A cocktail of mecillinam, a clinically approved antibiotic for use in humans that is not currently widely used, and cefsulodin or cephalexin could increase the efficacy of both while only using two antibiotics and minimizing drug interactions.

## Methods

### Growth conditions

Overnight cultures were grown at 37 °C in a shaking incubator in Luria Bertani (LB) broth (Fisher Scientific: 244620) or MOPS (Teknova: M2106) supplemented with 0.2% glycerol. For growth curves, a 1:1000 subculture of the overnight culture was made into fresh media and cells were grown shaking at 37 °C, unless otherwise noted. OD_600_ samples were taken every hour for the first 2 h and then every 30 min. In cases of increased starting inoculum uninoculated media was removed so that all flasks had the same starting volume.

### Slow growth conditions

MG1655 cells were inoculated 1:1000 from an overnight culture grown at 37 °C into LB and grown at 30 °C. OD_600_ measurements were taken every hour for the first 2 h and then every 30 min. Slow growth was tested by inoculating MG1655 cells 1:1000 from an overnight culture, grown in M63-lactose media (M63 salts + casamino acids + 0.25% lactose) into fresh M63-lactose media. Measurements were taken once every hour for 2 h before switching to measurements every 30 min. A22 (10 µg/ml) was added at the start of the growth curves or when cells reached an OD_600_ ~0.1.

### Spent media testing

Overnight spent media was created by growing wild-type cells in LB either overnight or to an OD_600_ ~0.1. About 5 ml of overnight culture was spun down at 5000×*g* for 10 min before filter (0.22 µm) sterilization. The OD_600_ 0.1 spent media was only filter sterilized. Overnight spent LB was mixed with fresh LB of the same batch to a final concentration of 10% before inoculation. 100% of the 0.1 spent LB was used. Cells were inoculated 1:1000 from an overnight culture of MG1655 cells. Both fresh LB and spent LB had A22 (10 µg/ml) added at the start of the growth curve and when the OD_600_ ~0.1. OD_600_ measurements were taken once every hour for 2 h before switching to measurements every 30 min.

### Increased cell density growth

Overnight cultures of MG1655 cells grown in LB were subcultured in 1:1000, 1:100, and 1:10 ratios into fresh LB. Cells were grown with and without A22 (10 µg/ml). OD_600_ measurements were taken every hour for 2 h before measuring every 30 min.

Overnight cultures of MG1655 cells were grown in MOPS (0.2% glycerol) before being spun down and resuspended in fresh MOPS (0.2% glycerol). The OD_600_ was measured and this was then used to calculate the amount of culture needed for a starting OD_600_ of 0.1, 0.05, 0.025, and 0.0125 with the same final volume. Each starting OD_600_ condition was grown in LB or LB + A22 (10 µg/ml). OD_600_ measurements were then taken every 30 min.

Growth rates were calculated using the following formula ${{{{{\rm{Growth}}}}}}\; {{{{{\rm{rate}}}}}}=\frac{{{{{{\rm{log }}}}}}\left({{{{{{\rm{O.D}}}}}}.}_{600}{{{{{\rm{T}}}}}}2\right)-{{{{{\rm{log }}}}}}({{{{{{\rm{O.D}}}}}}.}_{600}{{{{{\rm{T}}}}}}2)}{0.301({{{{{\rm{Hr}}}}}})}$. Time points were chosen based on log phase (linear growth on a log-scale plot) and at least 30 min after exposure to A22 (10 µg/ml). In order to average growth rates taken on different days we used the growth ratios $\frac{{{{{{\rm{Growth}}}}}}\; {{{{{\rm{rate}}}}}}\; {{{{{\rm{A}}}}}}22}{{{{{{\rm{Growth}}}}}}\; {{{{{\rm{rate}}}}}}\; {{{{{\rm{control}}}}}}}$ from each day and averaged across three days.

### Growth stage testing

Sterile LB was kept at 37 °C and culture of exponentially growing MG1655 cells at OD_600_ ~0.1 were subcultured into the pre-warmed media with and without A22 (10 µg/ml). The cells were diluted at 1:1, 1:10, and 1:100. OD_600_ measurements were taken every half hour post subculturing into pre-warmed media.

### Temperature shift

Overnight cultures of MG1655 cells and LMC582 (PBPts), grown at 30 °C, cells were subcultured 1:1000 into flasks with LB + 0.2% glycerol. Control flasks of each strain were kept at either 40 or 30 °C and not shifted over the course of the growth curve. Flasks were moved from the permissive to the nonpermissive temperature every hour. OD_600_ measurements were taken every hour for 2 h after which measurements were taken every 30 min.

Growth rates were calculated using the following formula ${{{{{\rm{Growth}}}}}}\; {{{{{\rm{rate}}}}}}=\frac{{{{{{\rm{log }}}}}}\left({{{{{{\rm{O.D}}}}}}.}_{600}{{{{{\rm{T}}}}}}2\right)-{{{{{\rm{log }}}}}}({{{{{{\rm{O.D}}}}}}.}_{600}{{{{{\rm{T}}}}}}2)}{0.301({{{{{\rm{Hr}}}}}})}$. Time points were chosen based on log phase and at least 30 minutes after temperature shift. To average growth rates taken on different days we used growth ratios $\frac{{{{{{\rm{Growth}}}}}}\,{{{{{\rm{rate}}}}}}\,{{{{{\rm{at}}}}}}\,{30}\;^{\circ }{{{{{\rm{C}}}}}}}{{{{{{\rm{Growth}}}}}}\,{{{{{\rm{rate}}}}}}\,{{{{{\rm{at}}}}}}\,{40}\;^{\circ }{{{{{\rm{C}}}}}}}$ and averaged across three days. Because cells shifted at T1 and T2 were shifted before reaching exponential growth, we used the 30 °C control growth rates.

### Deletion strains

All deletions were made via P1 lysate transduction from Keio deletion strains into MG1655 background. Transductants were selected on kanamycin plates and deletions were verified by PCR confirmation. A complete list of stains can be found in Table S1.

### Microscopy

#### Growth conditions

MG1655 cells were diluted 1:1000 from an overnight culture in fresh LB at 37 °C. Cells treated with A22 (10 µg/ml) had the antibiotic added at 1 h of growth. These cells were imaged at 1 h of growth, 3 h of growth, and 5 h of growth. In a separate experiment, cells were grown for 3 h when A22 (10 µg/ml) or mecillinam (3 µg/ml) was added to the media. These cells were then imaged at 3, 4, and 6 h.

#### Image acquisition

All imaging was done on M63 glucose 1% agarose pads at room temperature. Heat-killed cells were placed in a boiling water bath for 10 min prior to imaging. Phase-contrast images were collected on a Nikon Ni-E epifluorescent microscope equipped with a 100X/1.45 NA objective (Nikon), Zyla 4.2 plus cooled sCMOS camera (Andor), and NIS Elements software (Nikon).

#### Time lapse

MG1655 cells were inoculated at an OD_600_ ~0.01 and grown for 4 h in LB + A22 (10 µg/ml) until they displayed vesicles. Cells were then applied to M63 glucose + A22 (10 µg/ml) 1% agarose pads and the coverslip was sealed with paraffin wax. Cells were imaged every 5 min for 8 h in order to monitor cell lysis.

### Microscopy analysis

Cell contours were found using the MATLAB program Morphometrics (Ursell, Lee, et al. 2017). The coefficient of variation of intracellular diameter deviation and cell area (µm^2^) were determined in MATLAB using the contours from Morphometrics as previously described in refs. ^13,69^. Statistical significance was determined via ANOVA and Tukey’s HSD. All group comparisons not marked not significant are significant to a *P* value of 0.0001.

### Electron microscopy

#### Sample preparation and growth

MG1655 cells were grown from a starting OD_600_ ~0.01 in LB with A22 (10 µg/ml) for 4 h before being fixed with glutaraldehyde and osmium tetroxide. The sample was infiltrated with 1:1 ETOH/LRWhite and embedded in Polybed 812. The sample was sectioned at 70 nm, mounted to a carbon fiber grid, and stained with uranium acetate and lead citrate.

#### Image acquisition

Images of cells displaying vesicles were collected using a JEOL 2100 HRTEM operating at 200 kV.

### Keio library screen

Cells were grown in 96-well plates from Keio deletion library plates. Overnight plate cultures were inoculated 1:100 into LB plates and A22 (1 µg/ml) plates and grown overnight. The ratio of LB OD_600_ and A22 OD_600_ were averaged across three repetitions and those strains with a ratio of 4 or higher were considered to be more sensitive to A22.

### Statistics and reproducibility

All experiments were performed in triplicate on separate days. Supplemental Tables 2 and 4 indicate the total number of cells from the triplicate experiments in Figs. 1 and 2 were used to calculate cell area and IDD. Statistical tests were performed as indicated.

### Reporting summary

Further information on research design is available in the Nature Research Reporting Summary linked to this article.

## Supplementary information

Supplementary Information

Description of Additional Supplementary Files

Supplementary Data 1

Reporting Summary

## Data Availability

Any remaining information can be obtained from the corresponding author upon reasonable request.
