# Supplementary material for: Cell density-dependent antibiotic tolerance to inhibition of the elongation machinery requires fully functional PBP1B
Source: Commun Biol. 2022 Feb 3;5:107. doi: 10.1038/s42003-022-03056-x (PMC8813938; doi:10.1038/s42003-022-03056-x)
Supplement: Supplementary file 1 — Supplementary Information [file 42003_2022_3056_MOESM1_ESM.pdf]

Strain Name	Relevant Genotype	Source
MG1655	F- lambda- *ilvG- rfb-50 rph-1*	*E. coli* Genetic Stock Center
RS1	MG1655 *envC::kan*	This Study
RS2	MG1655 *yccK::kan*	This Study
LMC582	F- araD139 Δ(argF-lac)U169 deoC1 flbB5301 ptsF25 pbpa137(ts)	(Woldringh 1988)
LMC882	his, purB, proA, thi, lacY, rpsL, rodA(Ts)-52, zbe::Tn10	(Matsuzawa, Hayakawa et al. 1973)
ΔPBP1B	MG1655 *mrcB::kan*	This Study
ΔPBP1A	MG1655 *mrcA::kan*	This Study
Δ*yfeW*	MG1655 *yfew::kan*	This Study
Δ*murR*	MG1655 murR::kan	This Study
Δ*murP*	MG1655 murP::kan	This Study
Δ*murQ*	MG1655 murQ::kan	This Study
DR7V2	MG1655 *mrcB::frt pMB1 lacIqPtac kan*	(Ranjit, Jorgenson et al. 2017)
DR7N	MG1655 *mrcB::frt pMB1 lacIqPtac:: mrcB (G697C, T1528G) kan*	(Ranjit, Jorgenson et al. 2017)
DR7TP	MG1655 *mrcB::frt pMB1 lacIqPtac:: mrcB (G697C) kan*	(Ranjit, Jorgenson et al. 2017)
DR7GT	MG1655 *mrcB::frt pMB1 lacIqPtac:: mrcB (T1528G) kan*	(Ranjit, Jorgenson et al. 2017)

P. aeruginosa strains

Strain Name	Relevant Genotype	Source
PA01	Wild Type	*Pseudomonas* Genetic Stock Center

S.Flexneri strains

Strain Name	Relevant Genotype	Source
ATCC 12022	Wild Type	ATCC

Table S1: *E. coli*, *P. aeruginosa*, and *S. flexneri* strains.

	Cells used for quantification		
	LB	T1 A22	T3 A22
T1	2006	N/A	N/A
T2	1193	796	N/A
T3	1418	373	N/A
T4	948	264	1283
T5	580	394	854
T6	1432	441	1028
T7	2053	561	902

Table S2: The number of cells used for quantification for Fig. 1 C, D. NA- not applicable

Starting O.D.$_{600}$ repetition	MOPS growth rate (generations/hour)	A22 growth rate (generations/hour)	A22 Growth Rate/MOPS Growth Rate
0.1 Rep 1	1.42	1.36	0.96
0.1 Rep 2	1.57	1.60	1.02
0.1 Rep 3	1.63	1.66	1.01
0.05 Rep 1	1.56	1.44	0.93
0.05 Rep 2	1.49	1.40	0.94
0.05 Rep 3	1.59	1.59	1.00
0.025 Rep 1	1.64	1.32	0.80
0.025 Rep 2	1.60	1.48	0.92
0.025 Rep 3	1.76	1.20	0.68
0.0125 Rep 1	1.72	1.16	0.68
0.0125 Rep 2	1.87	1.33	0.71
0.0125 Rep 3	1.83	1.25	0.60

Table S3: Raw growth rates and ratios. Growth rates of every repetition of the experiment in Fig. 2C (generations/hour) and their A22 Growth Rate/MOPS Growth Rate within a starting O.D.

	Cells used for quantification			
	0.01 LB	0.01 A22	0.1 LB	0.1 A22
T1	210	353	723	731
T2	331	407	556	482
T3	516	186	782	417
T4	946	205	1171	457

Table S4: The number of cells used for quantification in Fig. 2 E,F.

a

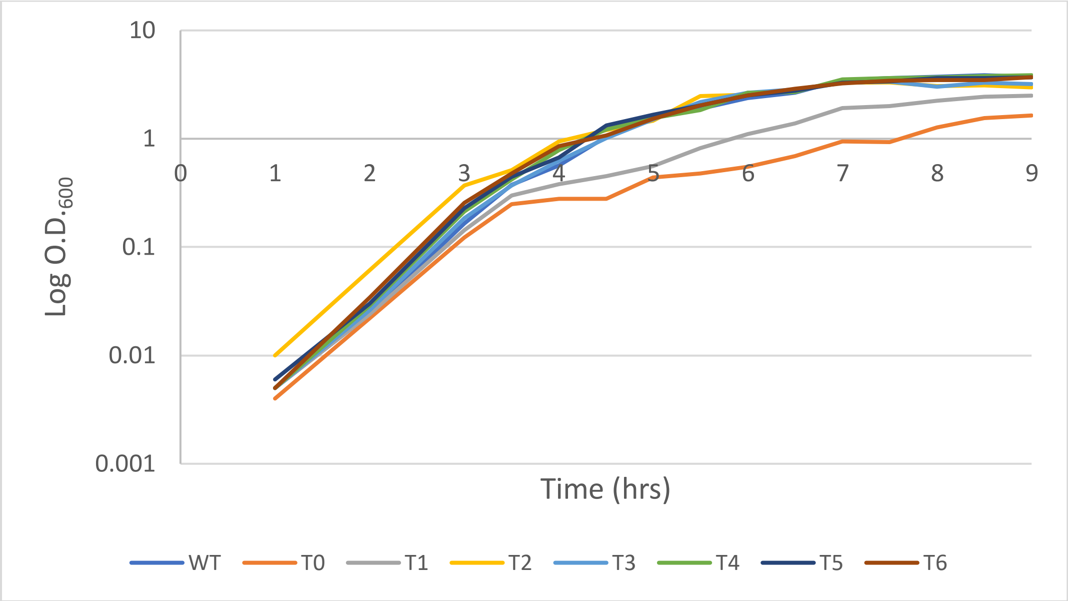

b

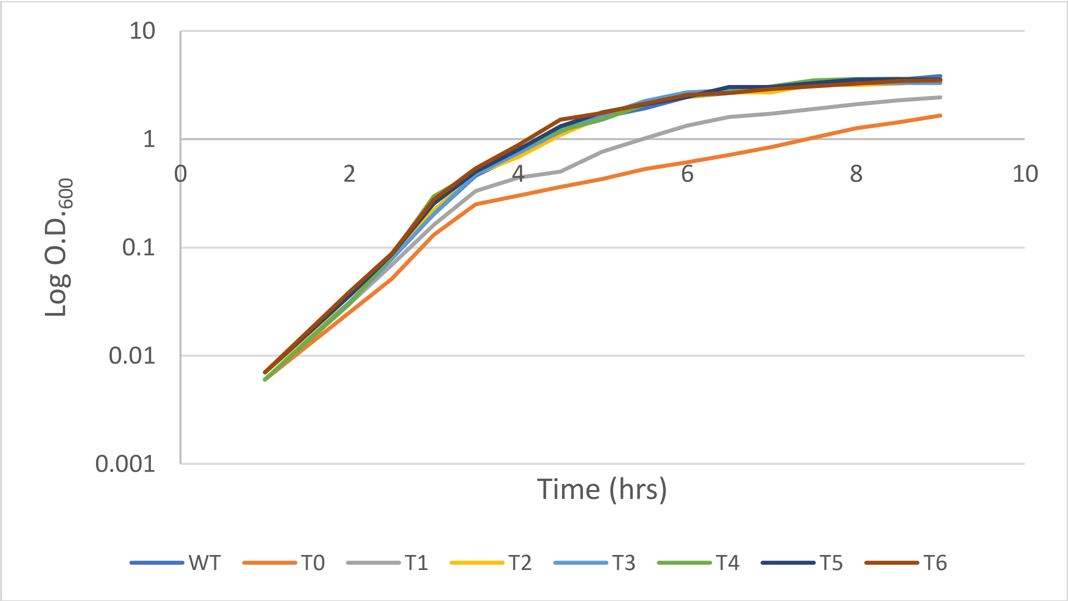

Figure S1. **Additional repetitions of A22 time course growth curve.** (ab) WT cells at 37 ºC with A22 (10 µg/ml) added every hour from 0 to 6 hours.

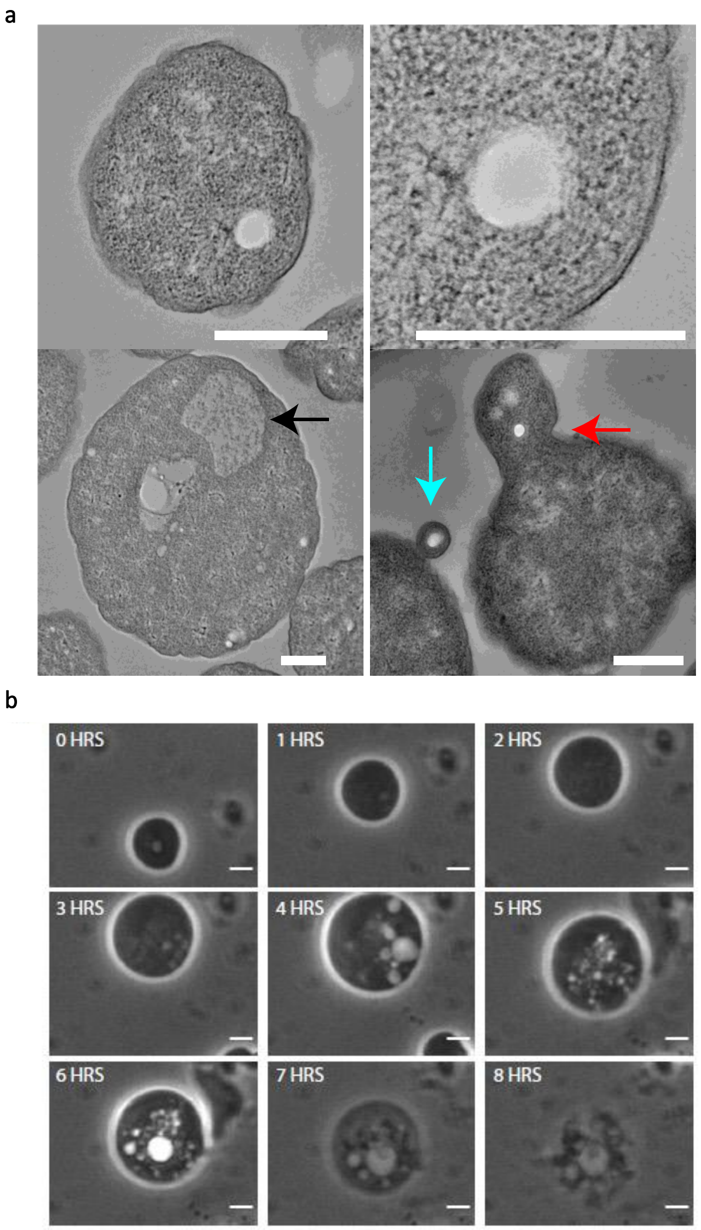

Figure S2. **Sensitive cells display diverse vesicles and are a sign of cell stress.** (a) Thin sectioned (70 nm) TEM of WT cells grown from an initial O.D.$_{600}$ ~0.01 in LB with A22 (10 µg/ml) for 4 hours. Scale bars are equal to 500 nm. Empty vesicle example-white arrow. Filled vesicle example-black arrow, membrane bleb example-blue arrow, and asymmetrical division example-red arrow. (b) Time lapse phase contrast images of A WT cell grown from an initial O.D.$_{600}$ ~0.01 in LB with A22 (10 µg/ml) for 4 hours before being grown on M63 glucose and A22 (10 µg/ml) 1% agarose pads at 25 ºC for 8 hours. Images were taken every 5 min for 8 hours. Scale bars represent 2 µm.

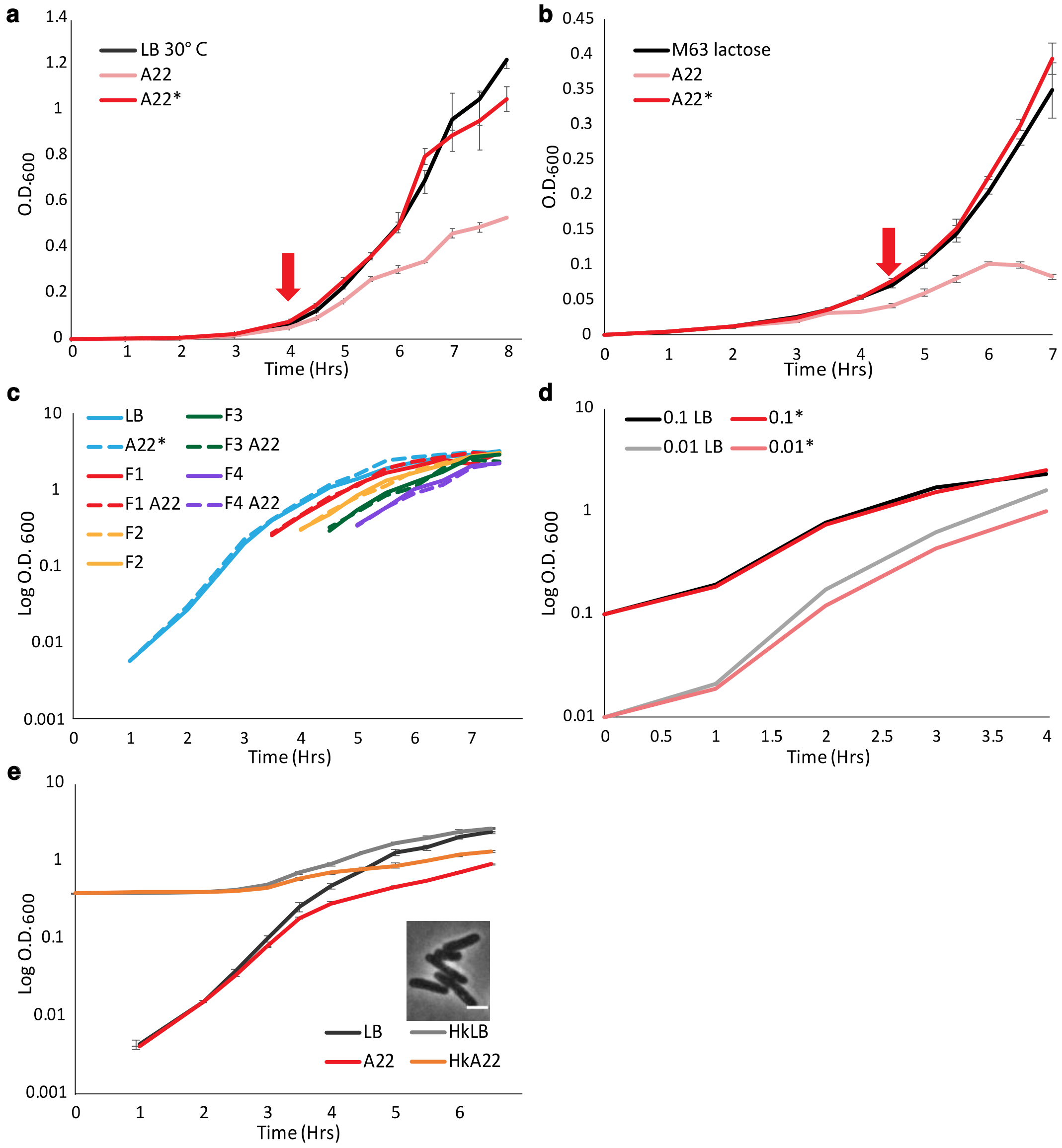

Figure S3. **Density dependent growth resistance is not caused by slow growth or limited mass doublings** (a) Growth curve of WT cells grown at 30° C in LB without A22, LB_{A22} (10 µg/ml), and A22 added at the indicated time where O.D.600 ~0.1 (*). (b) Growth curve of WT cells in M63 lactose media grown at 37 ºC in LB, LB_{A22} (10 µg/ml), and A22 added at the indicated time where O.D.600 ~0.1 (*). (c) Representative growth curve of WT cells. Cells were grown at 37 ºC for three hours and spiked with A22 (10 µg/ml) then inoculated at a 1:1 ratio into prewarmed LB and LB_{A22} (F1 and F1 A22). These cells were then grown for half an hour before repeating this procedure three more times. (F2-F4). (d) growth curve of WT cells treated use for imaging in Fig. 2d. (e) Representative growth curve of WT cells with or without A22. Heat-killed (Hk) cells were added at an $O.D._{600}$ 0.4. Inset shows heat-killed cells are still intact.

a

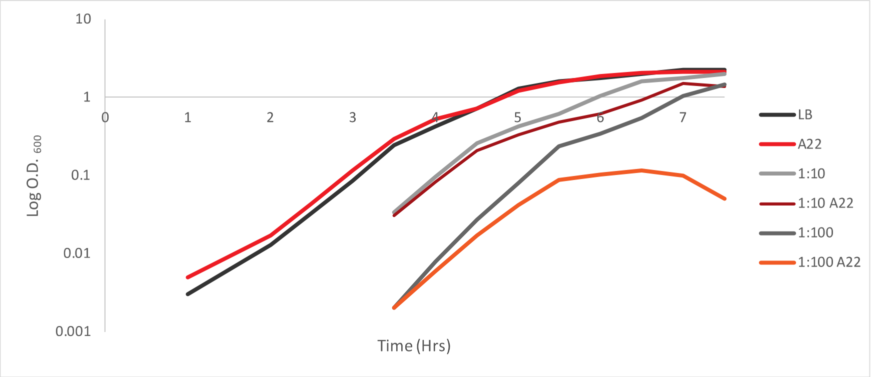

b

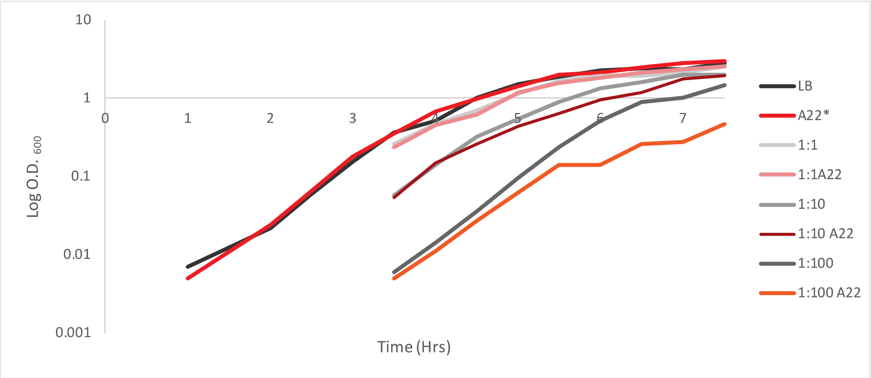

Figure S4. **Additional repetitions of pre-warmed back dilution growth curve.** (ab) Log-scale growth curve of WT cells grown at 37 ºC with different inoculation dilutions. Cells were grown with and without A22 (10 µg/ml) in LB at the indicated initial inoculation ratio from overnight cells.

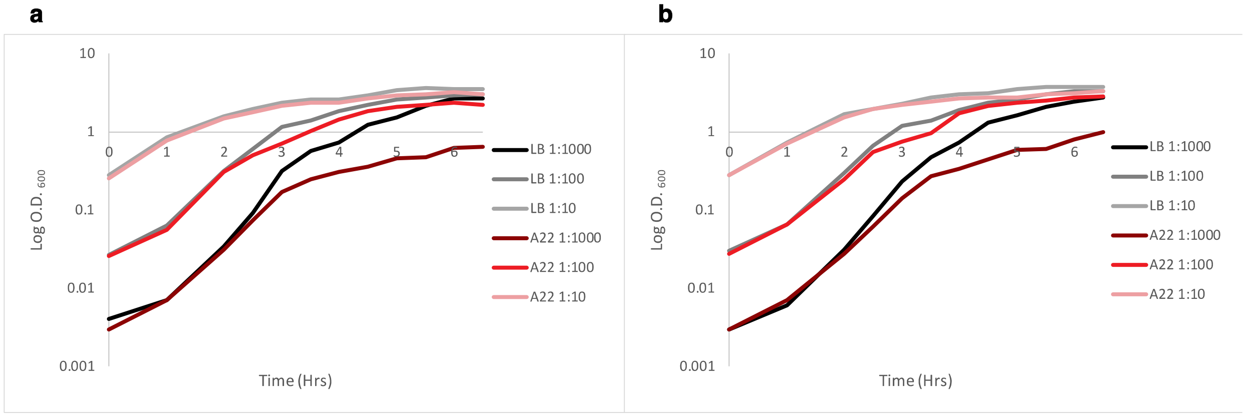

Figure S5. **Additional repetitions of starting dilution growth curves.** (ab) Log-scale growth curve of WT cells grown at 37 ºC with different inoculation dilutions. Cells were grown with and without A22 (10 µg/ml) in LB at the indicated initial inoculation ratio from overnight cells.

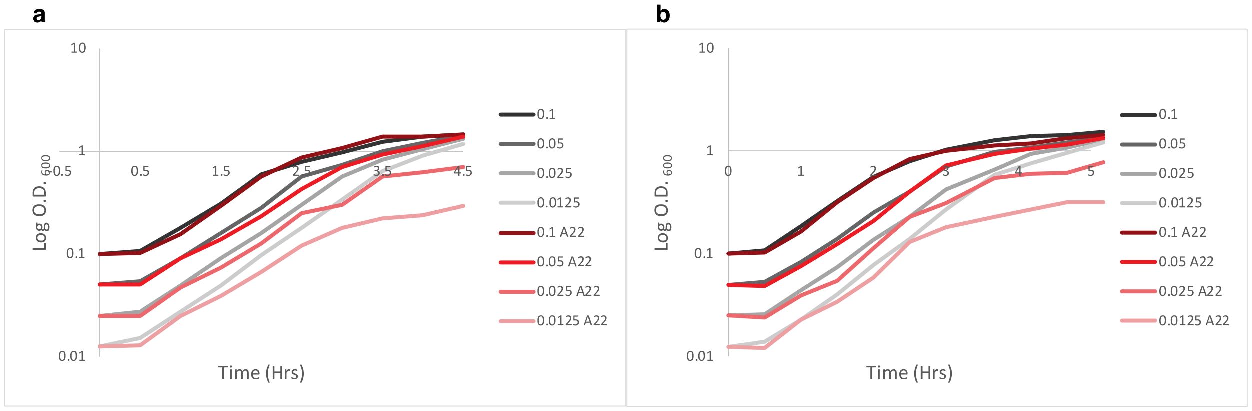

Figure S6. **Additional repetitions of starting O.D. growth curves.** (ab) log-scale growth curve of wild type cells with different starting O.D.s. Overnight cells were washed and resuspended into new MOPS media and inoculated at the indicated $O.D._{600}$ with and without A22 (10 µg/ml).

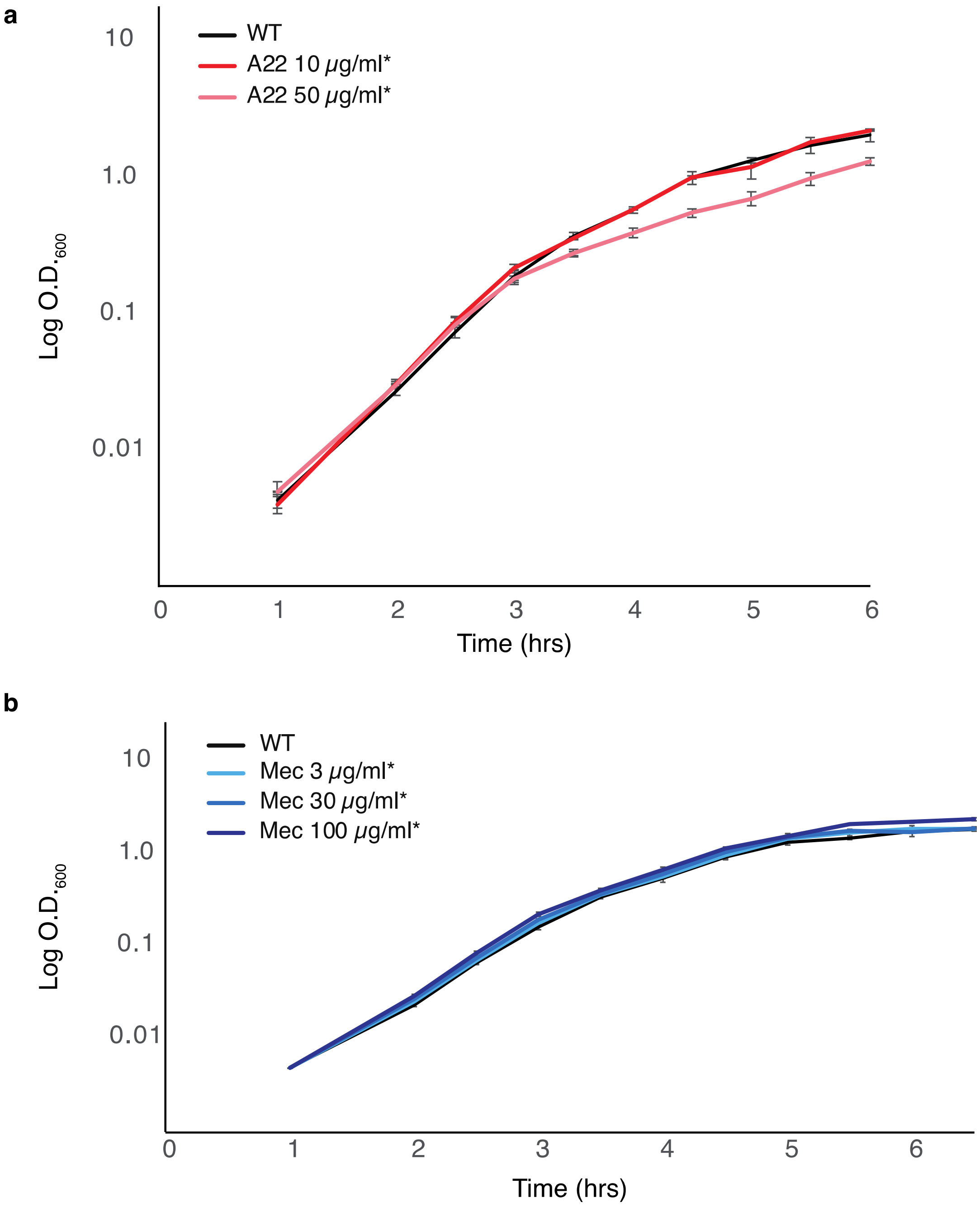

Figure S7. **Increased drug concentrations do not remove DDGR**. (a) Growth curve of WT cells grown at 37 ºC without A22, A22 (10 µg/ml) or A22 (50 µg/ml) added at $O.D._{600}$ ~0.1 (*)(~T3). (b) Growth curve of wild type cells grown at 37 ºC without mecillinam, mecillinam (3 µg/ml), mecillinam (30 µg/ml), or mecillinam (100 µg/ml) added at $O.D._{600}$ ~0.1 (*)(~T3).

a

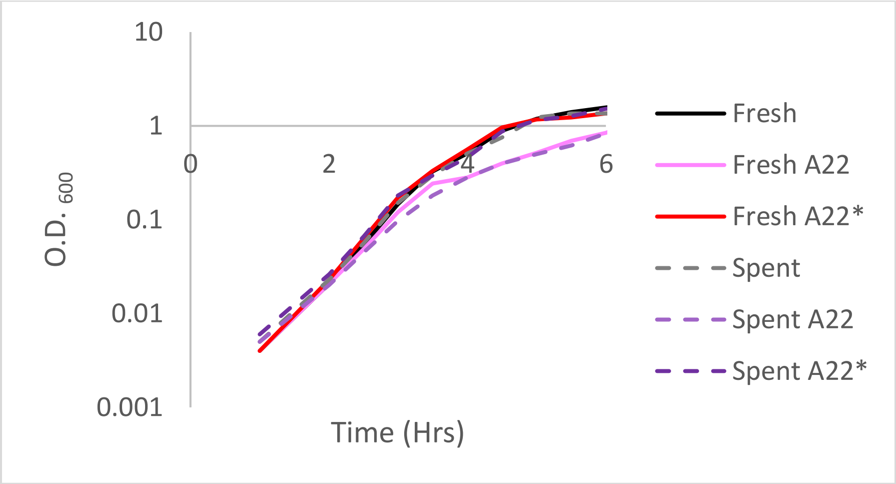
10
1
0.1
0.01
0.001
O.D. 600
0
2
4
6
Time (Hrs)
Fresh
Fresh A22
Fresh A22*
Spent
Spent A22
Spent A22*

b

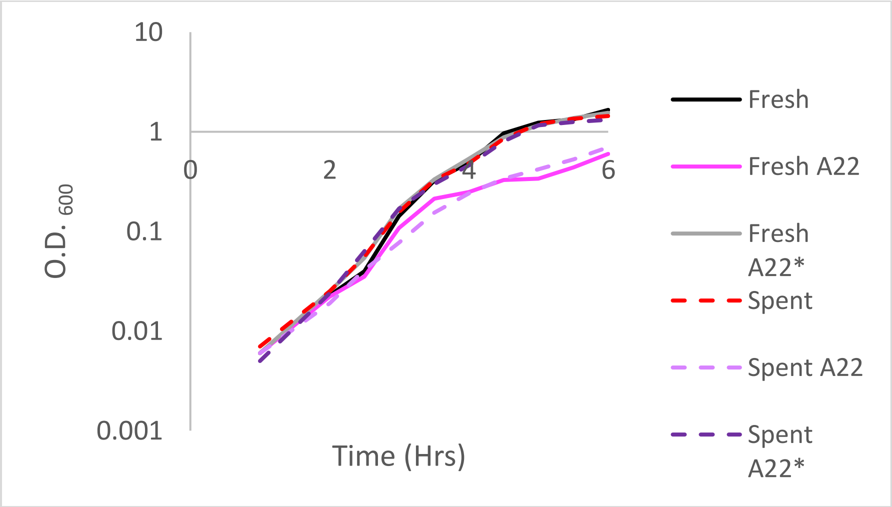
10
1
0.1
0.01
0.001
O.D. 600
0
2
4
6
Time (Hrs)
Fresh
Fresh A22
Fresh A22*
Spent
Spent A22
Spent A22*

c

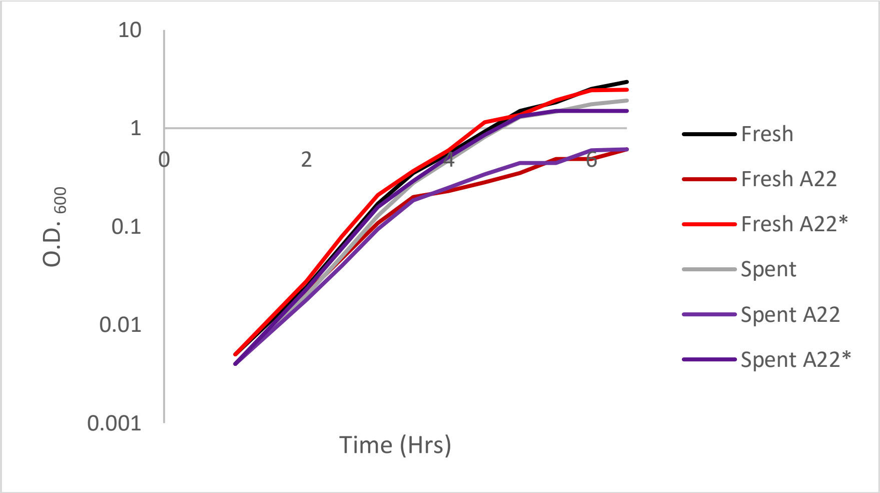
10
1
0.1
0.01
0.001
O.D. 600
0
2
4
6
Time (Hrs)
Fresh
Fresh A22
Fresh A22*
Spent
Spent A22
Spent A22*

d

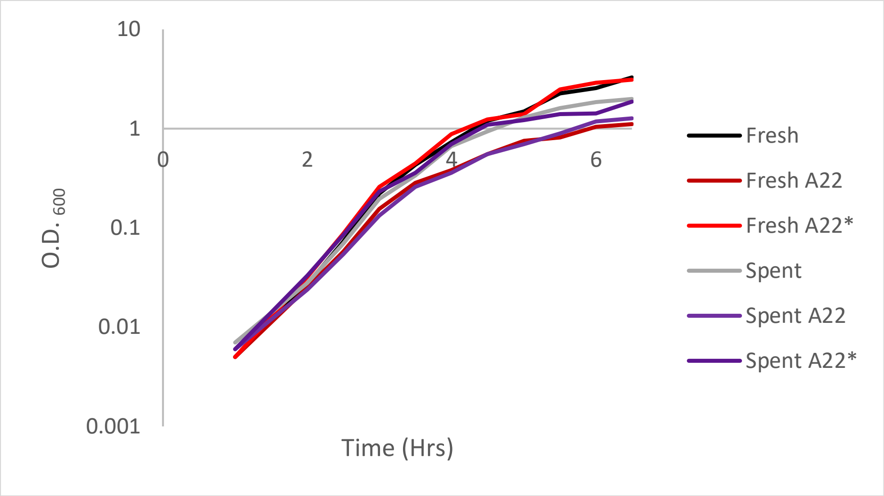

Figure S8. **Additional repetitions of spent media growth curves.** (ab) Growth curves of WT cells grown at 37 ºC with 100% spent media from a culture grown to an O.D.600 0.1. Cells were grown in LB, A22 (10 µg/ml), and A22 added at O.D.600 ~0.1 (*). (cd) Growth curves of WT cells grown at 37 ºC with 10% spent media from an overnight culture. Cells were grown in LB, A22 (10 µg/ml), and A22 added at O.D.600 ~0.1 (*).

a

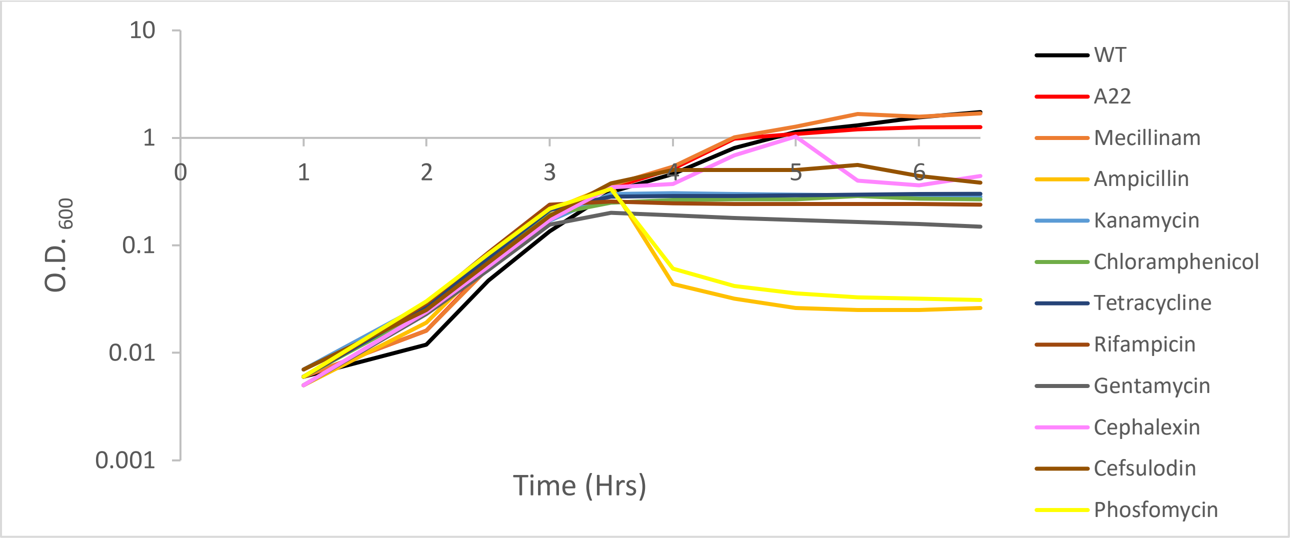

b

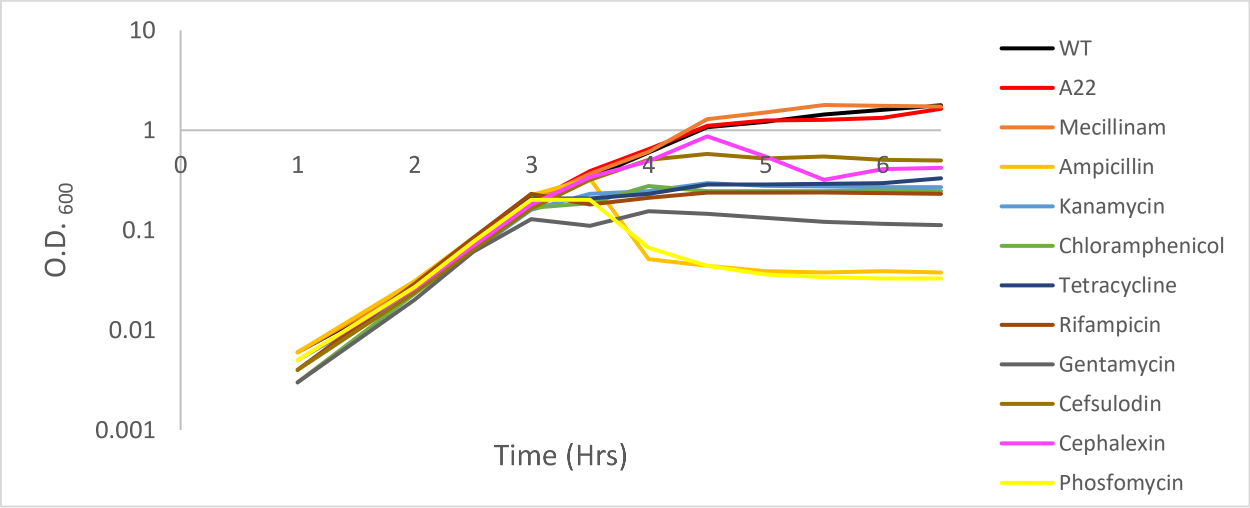

Figure S9. **Additional repetitions of antibiotic spike growth curve.** (ab) Growth curve of WT cells grown at 37 ºC treated with a variety of antibiotics at T3. A22 (10 µg/ml), cephalexin (10 µg/ml), mecillinam (3 µg/ml), ampicillin (100 µg/ml), phosphomycin (10 µg/ml), cefsulodin (30 µg/ml), kanamycin (30 µg/ml), chloramphenicol (35 µg/ml), tetracycline (10 µg/ml), gentamycin (45 µg/ml), and rifampicin (500 µg/ml).

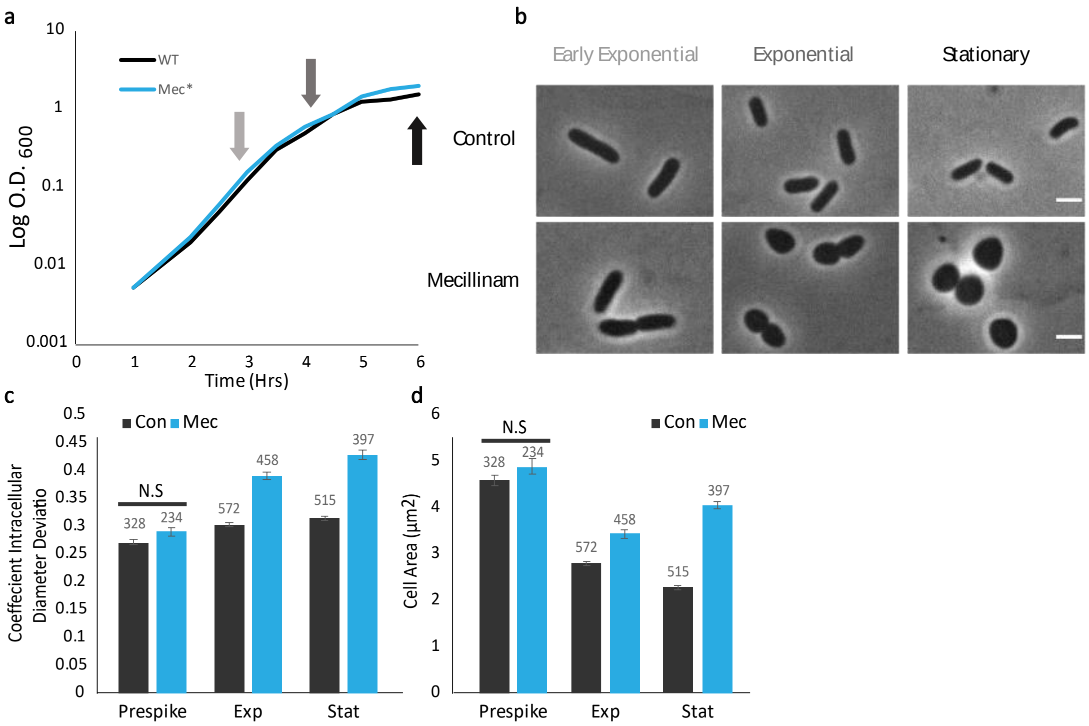

Figure S10. **Mecillinam is still active during DDGR** (a) Representative growth curve of WT cells grown at 37 ºC used for imaging. Arrows indicate times used for imaging. Mecillinam (3 µg/ml) was added at the first arrow concurrently with imaging. (b) Representative phase contrast imaging of WT cells at early exponential phase, exponential phase, and stationary phase with and without A22 addition at early exponential phase. Scale bar is equal to 2µm. (c) Pooled coefficient of intracellular diameter deviation from cell imaging. All comparisons not marked not significant are statistically significant P <0.001. (d) Pooled cell area (µm^2) from cell imaging. All comparisons not marked not significant are statistically significant P <0.001.

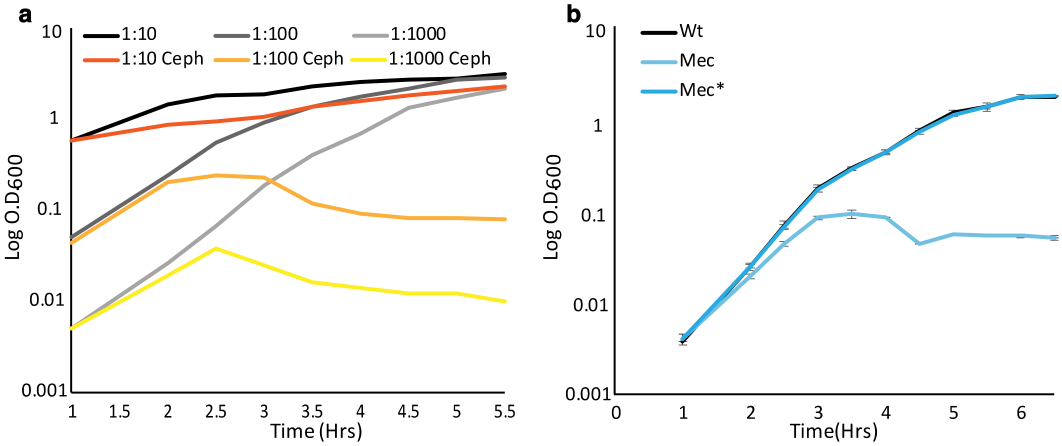

Figure S11. **Cephalexin does not display DDGR.** (a) Representative growth curve of WT cells grown at 37 ºC in LB and $LB_{cephalexin}$ (10µg/ml) inoculated at the following ratios of overnight culture to new LB 1:10, 1:100, 1:1000. (b) Growth curve of WT cells grown at 37 ºC in LB, $LB_{mecillinam}$ (3 µg/ml), and LB with mecillinam added at $O.D._{600}$ ~0.1 (T3).

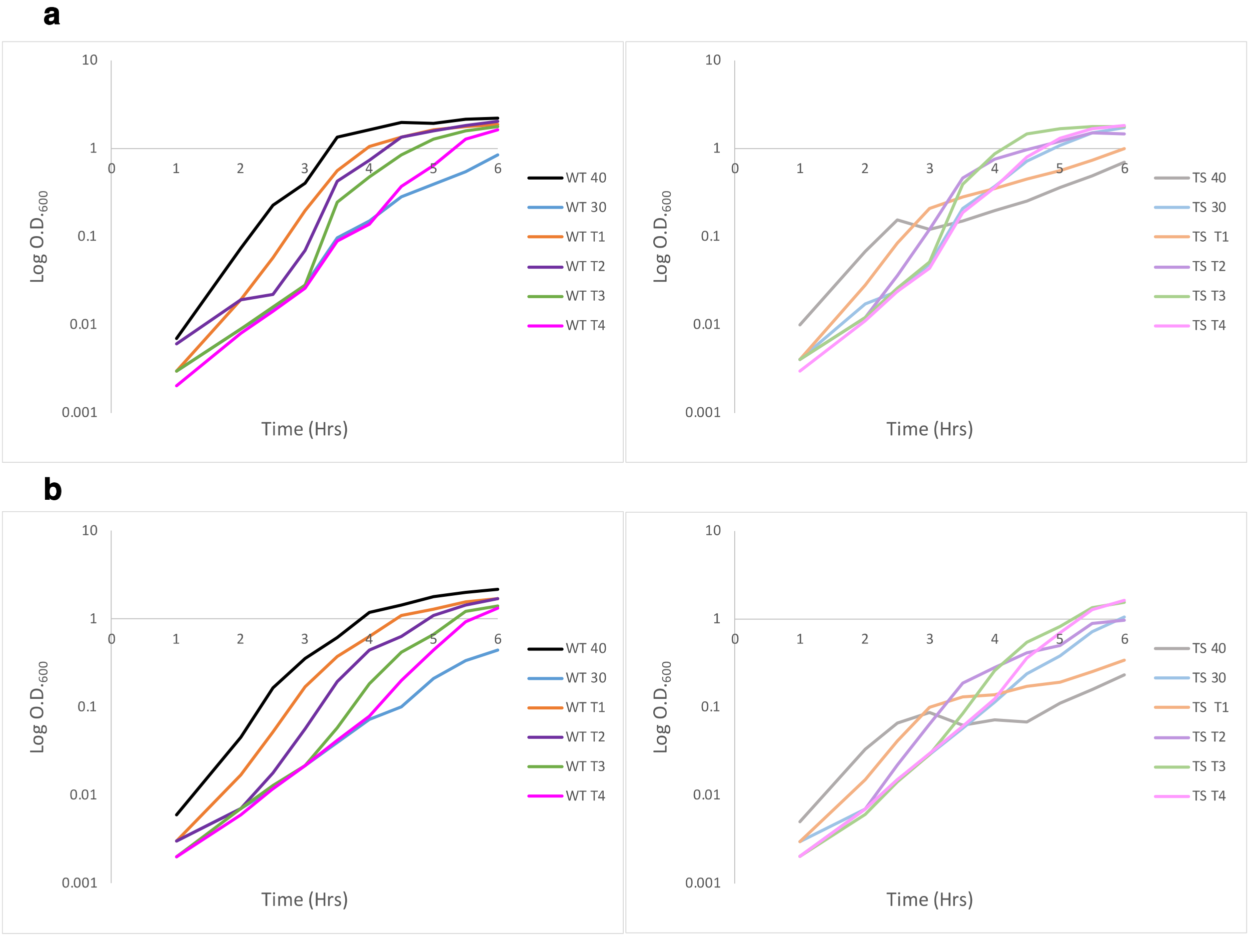

Figure S12. **Additional repetitions of temperature sensitive PBP2 growth curve.** (ab) growth curves of WT and PBP2ts cells grown in LB supplemented with glycerol at 40ºC, 30º C, and shifted from 30º C to 40º C every hour every hour for four hours.

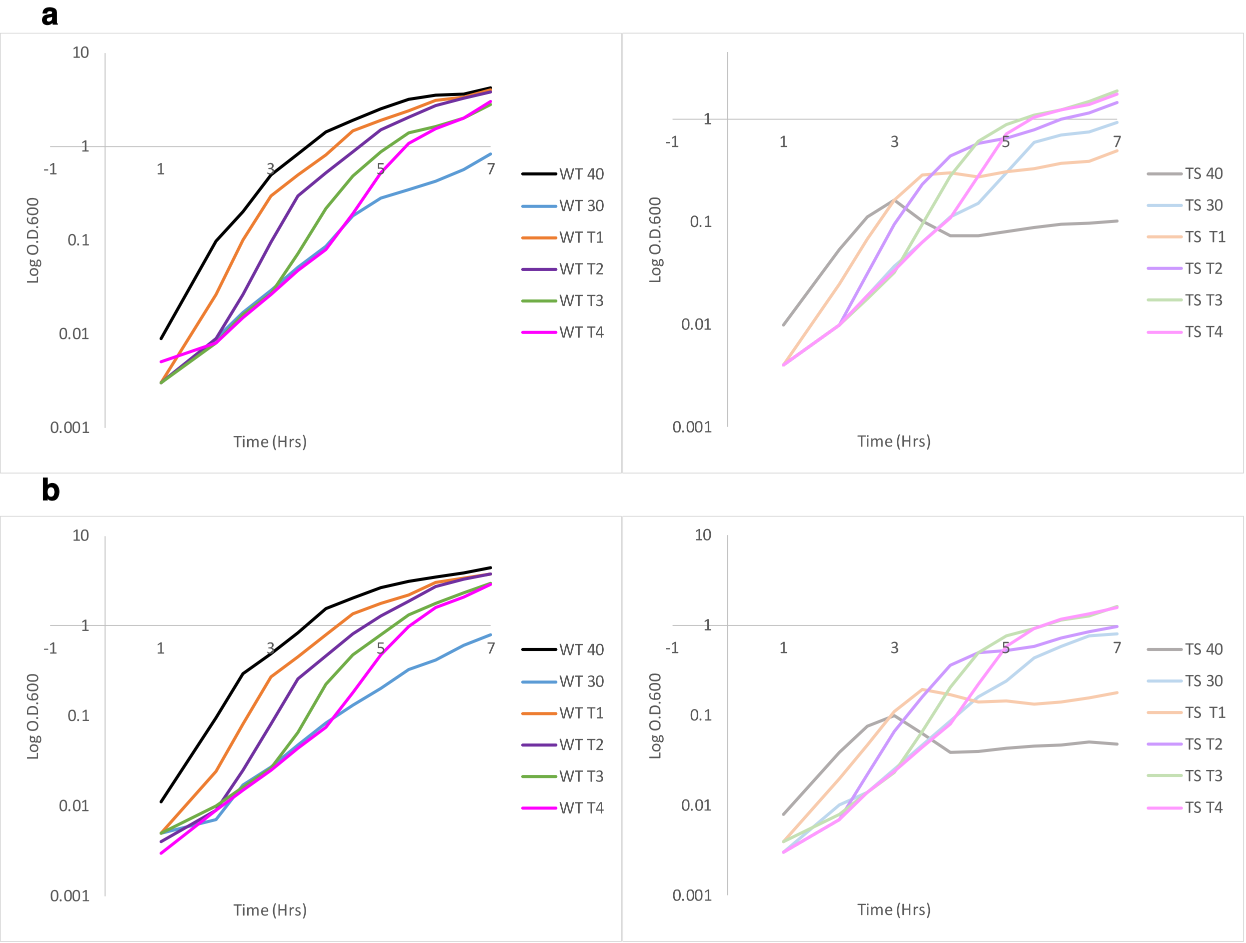

Figure S13. **Additional repetitions of temperature sensitive RodA growth curve.** (ab) growth curves of WT and RodAts cells grown in LB supplemented with glycerol at 40ºC, 30º C, and shifted from 30º C to 40º C every hour every hour for four hours.

a

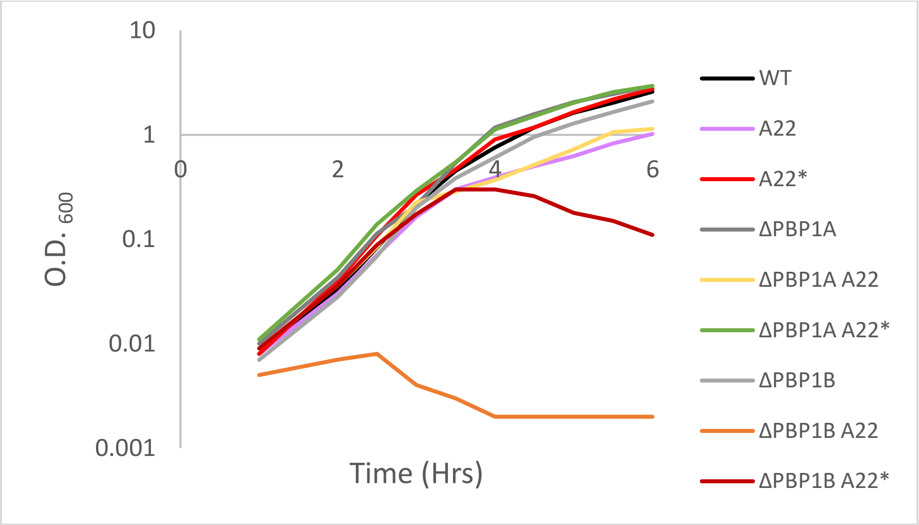

10
1
0.1
0.01
0.001
O.D. 600
0
2
4
6
Time (Hrs)
WT
A22
A22*
ΔPBP1A
ΔPBP1A A22
ΔPBP1A A22*
ΔPBP1B
ΔPBP1B A22
ΔPBP1B A22*

b

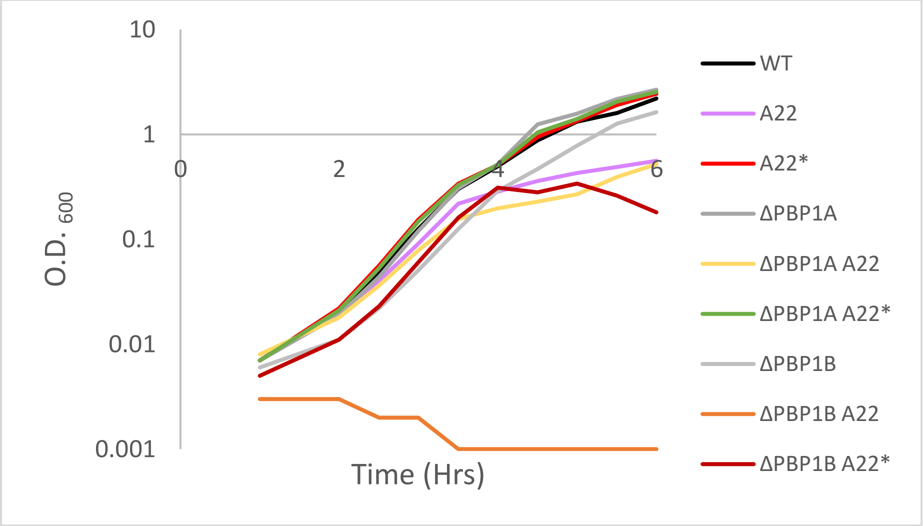

10
1
0.1
0.01
0.001
O.D. 600
0
2
4
6
Time (Hrs)
WT
A22
A22*
ΔPBP1A
ΔPBP1A A22
ΔPBP1A A22*
ΔPBP1B
ΔPBP1B A22
ΔPBP1B A22*

c

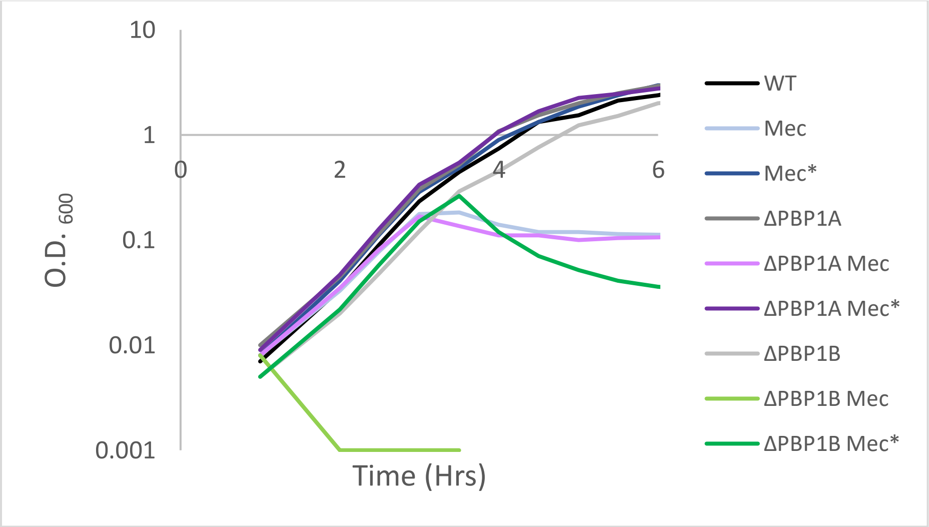

10
1
0.1
0.01
0.001
O.D. 600
0
2
4
6
Time (Hrs)
WT
Mec
Mec*
ΔPBP1A
ΔPBP1A Mec
ΔPBP1A Mec*
ΔPBP1B
ΔPBP1B Mec
ΔPBP1B Mec*

d

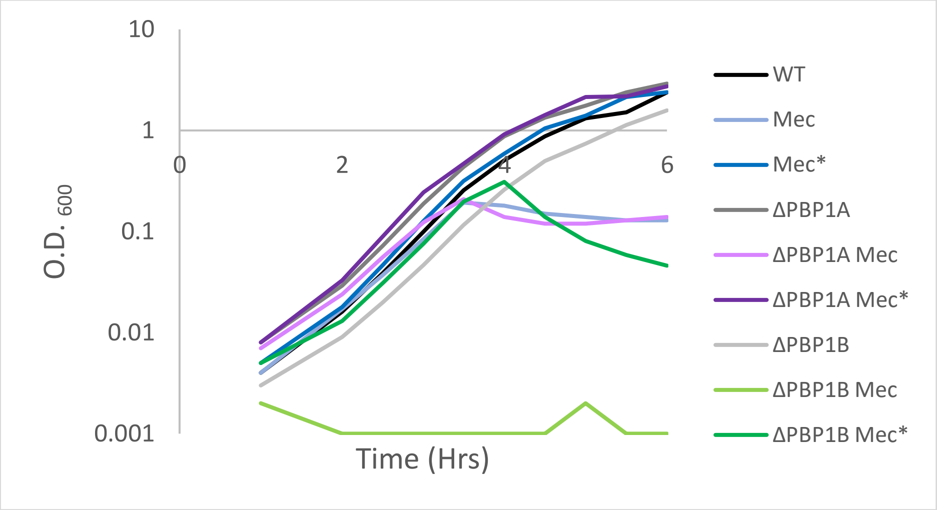

Figure S14. **Additional repetitions of ΔPBP1B and ΔPBP1A growth curves with A22 and mec.** (ab) Growth curves of WT, ΔPBP1A, and ΔPBP1B cells grown at 37 ºC in LB without A22, A22 (10 µg/ml), and A22 added at O.D.$_{600}$ ~0.1 (*). (cd) Representative growth curves of WT, ΔPBP1A, and ΔPBP1B grown at 37 ºC in LB without mecillinam, mecillinam (3 µg/ml), and mecillinam added at O.D.$_{600}$~0.1. (*).

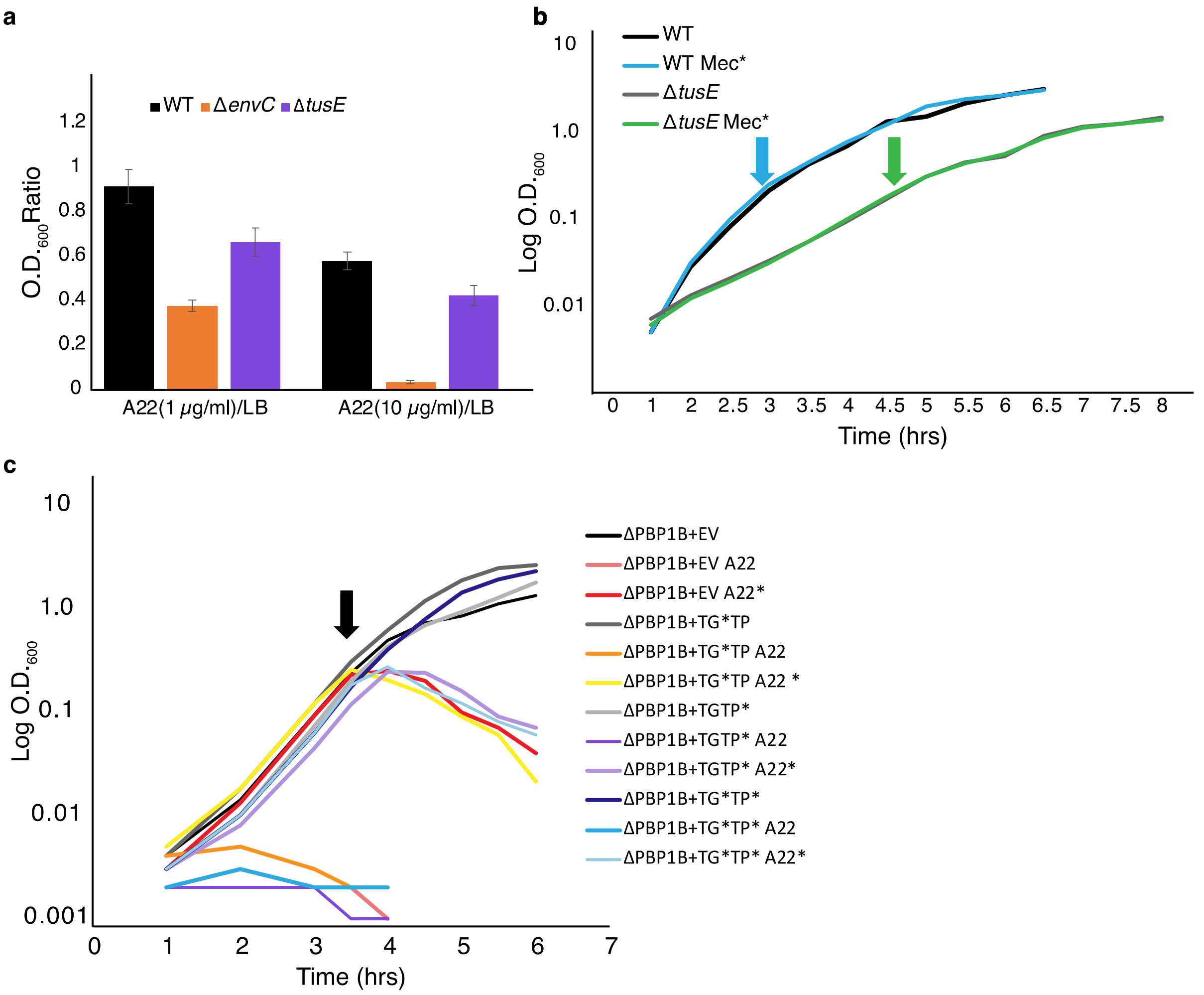

Figure S15. **Strain sensitive to A22 also display DDGR.** (a) Ratio of O.D.600 of strains grown in 1 µg/ml or 10 µg/ml A22 vs LB for 6 hrs. (b) Representative growth curve of wild-type and Δ*tusE* strains. Grown at 37 ºC without mecillinam, mecillinam (3 µg/ml), and mecillinam added at the indicated timepoint per strain where O.D.600 ~0.1. (c) Representative growth curve of DR7V2 empty vector (ΔPBP1B +EV), DR7GT nonfunctional transglycosylation (ΔPBP1B+TG*TP), DR7TP nonfunctional transpeptidation (ΔPBP1B+TGTP*), and DR7N with neither domain functioning (ΔPBP1B+TG*TP*) cells grown at 37 ºC in LB without A22, A22 (10 µg/ml), and A22 added at the indicated time where O.D.600 ~0.1 (*).

a

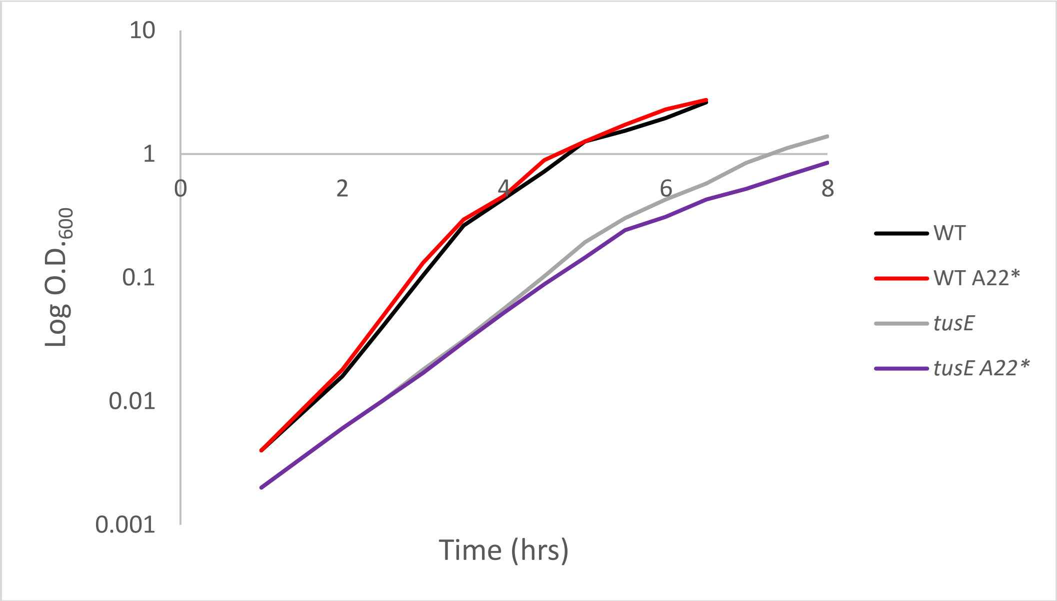

b

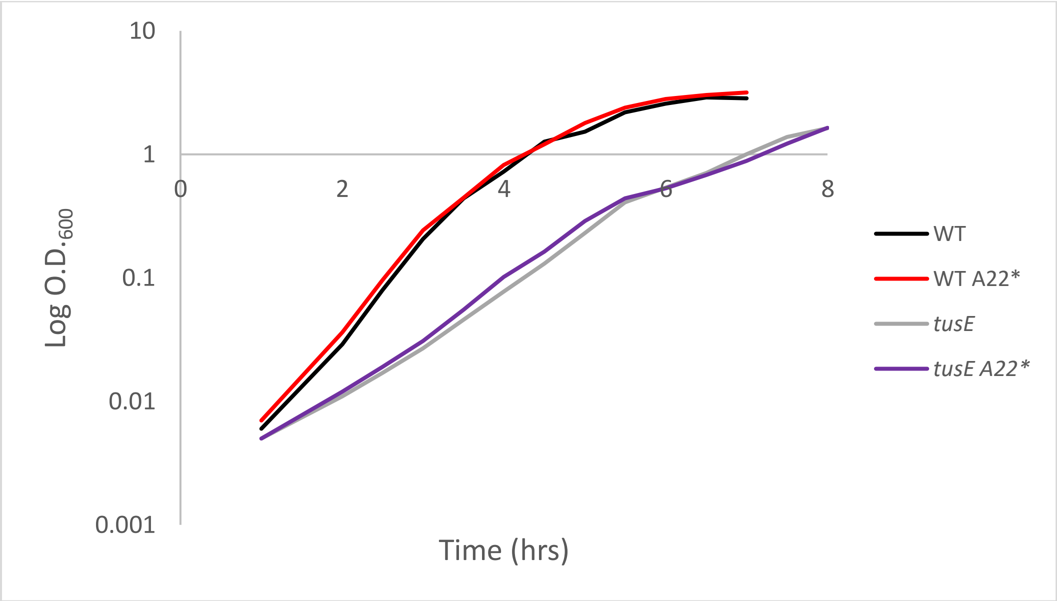

Figure S16. **Additional repetitions of Δ*tusE* A22 growth curves.** (ab) Representative growth curves of WT and Δ*tusE* cells grown at 37 ºC in LB without A22 and A22 (10 μg/ml) added at the time per strain where $O.D._{600}$ ~0.1. (*).

a

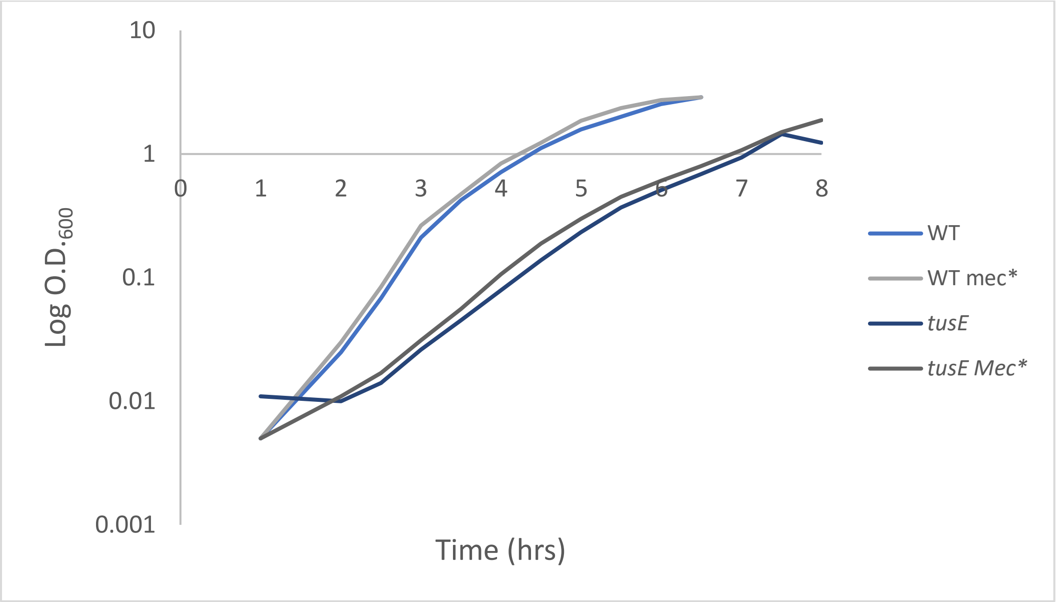

b

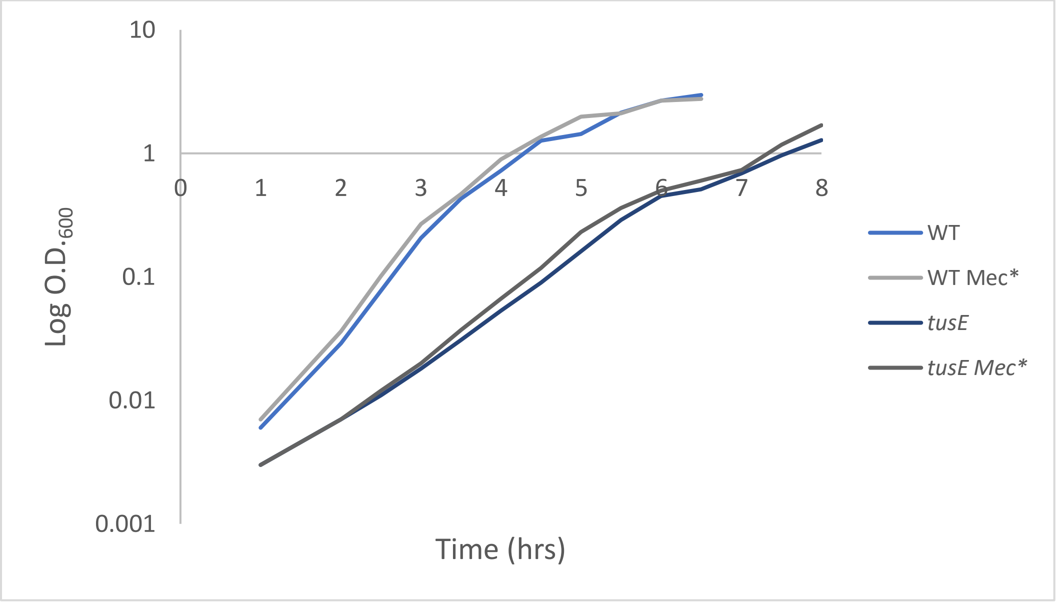

Figure S17. **Additional repetitions of Δ*tusE* mec growth curves.** (ab) Representative growth curves of WT and Δ*tusE* cells grown at 37 ºC in LB without mecillinam (mec) or mec (3 μg/ml) added at the time per strain where $O.D._{600}$ ~0.1. (*).

a

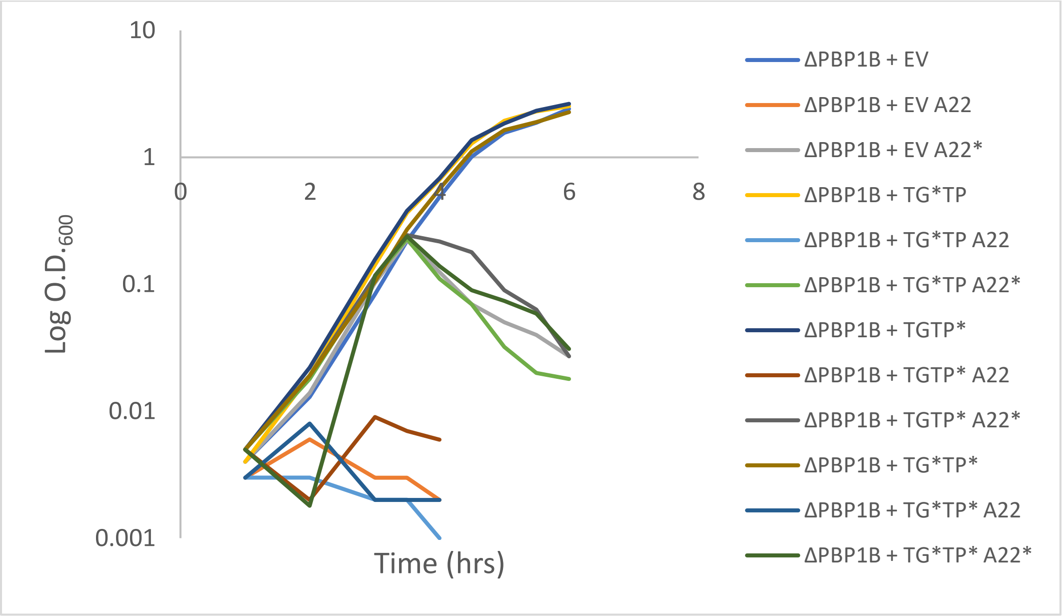

b

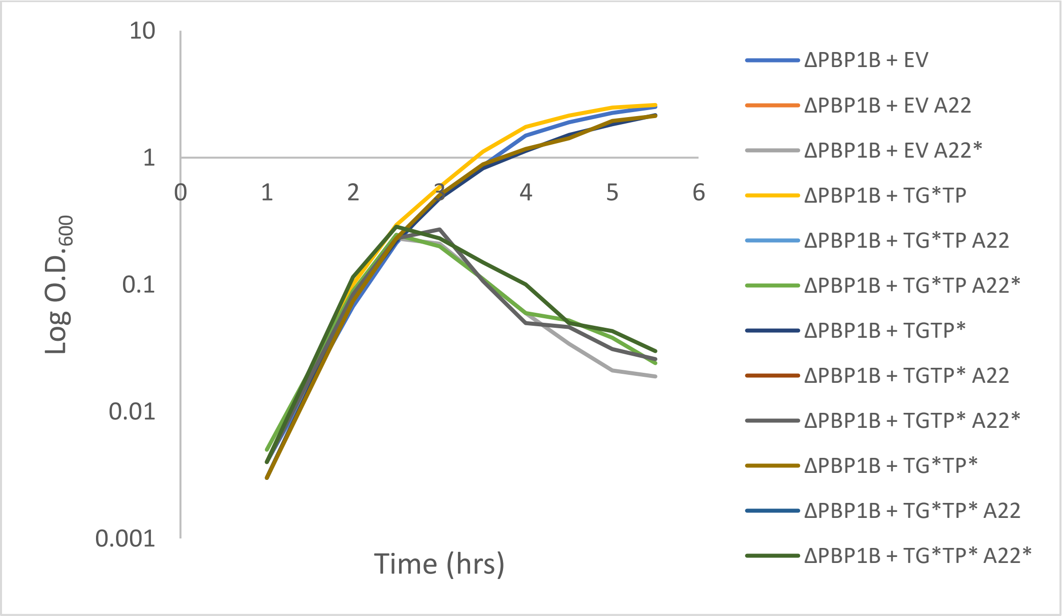

Figure S18. **Additional repetitions of PBP1B domain mutations growth curve.** (ab) growth curves of DR7V2 (ΔPBP1B +EV), DR7GT (ΔPBP1B+TG*TP), DR7TP (ΔPBP1B+TGTP*), and DR7N (ΔPBP1B+TG*TP*) cells grown at 37 ºC in LB without A22, A22 (10 µg/ml), or A22 added O.D.$_{600}$ ~0.1 (*).

a

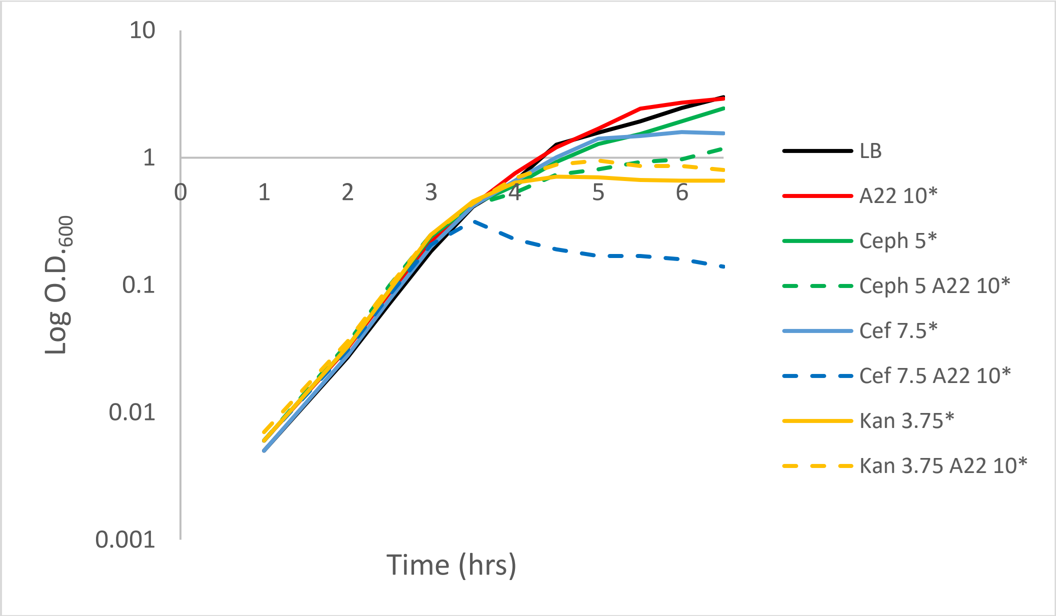

b

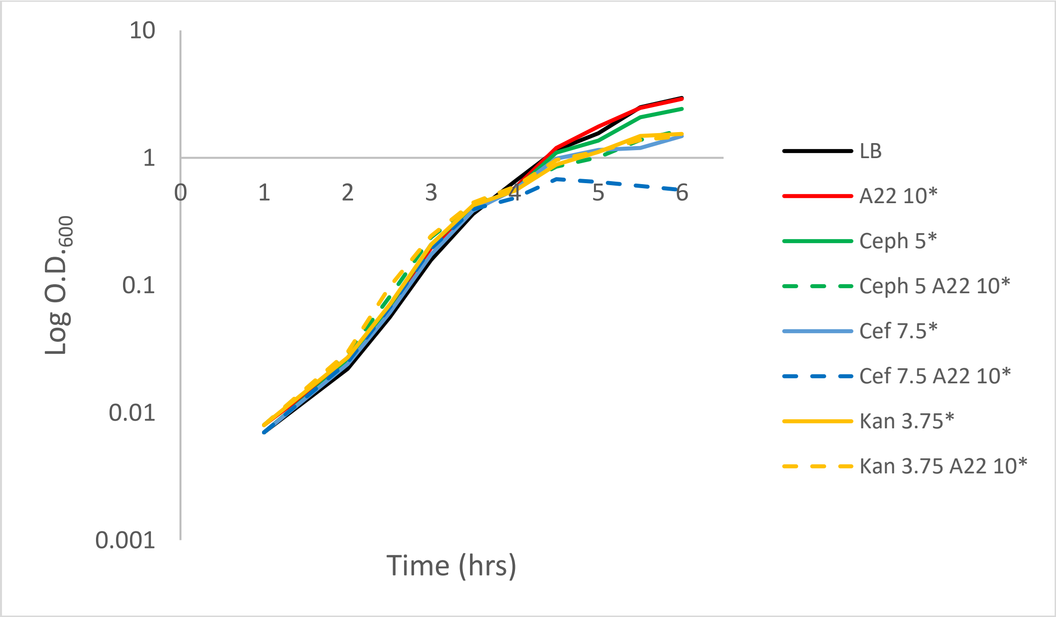

Figure S19. **Additional repetitions of antibiotic combination growth curves.** (ab) Growth curves of WT cells grown at 37 ºC in LB without antibiotics and the indicated antibiotics at the concentrations (µg/ml) added at $O.D._{600}$ ~0.1.

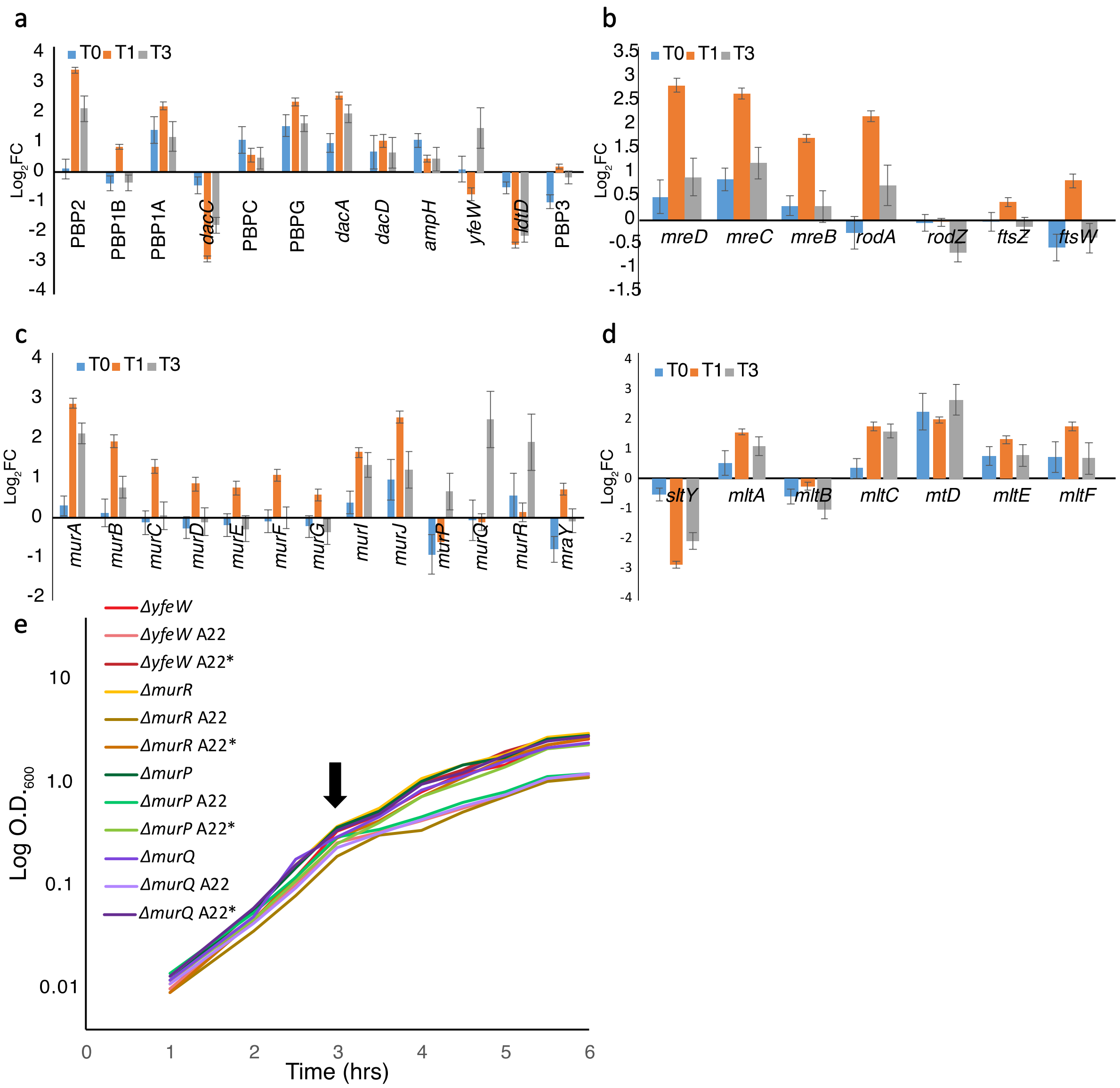

Figure S20. **Transcriptional response of cell wall synthesis and recycling genes.** (a-d) The Log_2 fold change of transcripts of indicated genes from E. coli BW25113 in LB. Transcripts were compared between indicated time points and an overnight culture. (a) PBP transcript levels. (b) Divisome and elongasome transcript levels. (c) Mur family transcripts levels. (d) mlt transcripts levels. (e) Representative growth curve of Δ*yfeW*, Δ*murR*, Δ*murP*, and Δ*murQ* cells grown at 37 ºC in LB without A22, A22 (10 µg/ml), and A22 added at the indicated time where $O.D._{600}$ ~0.1 (*).

a

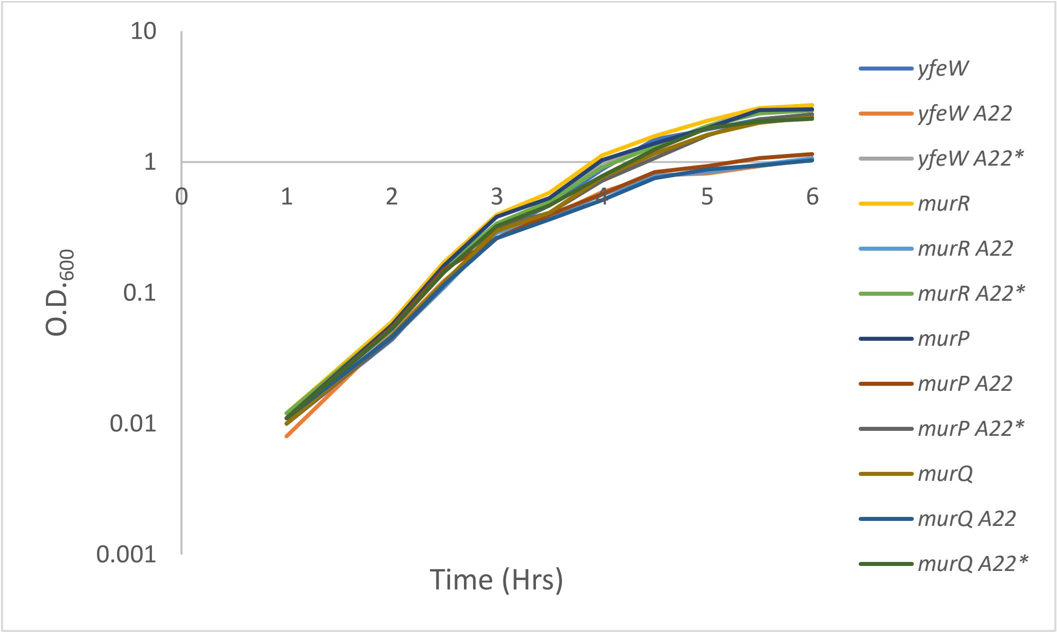

b

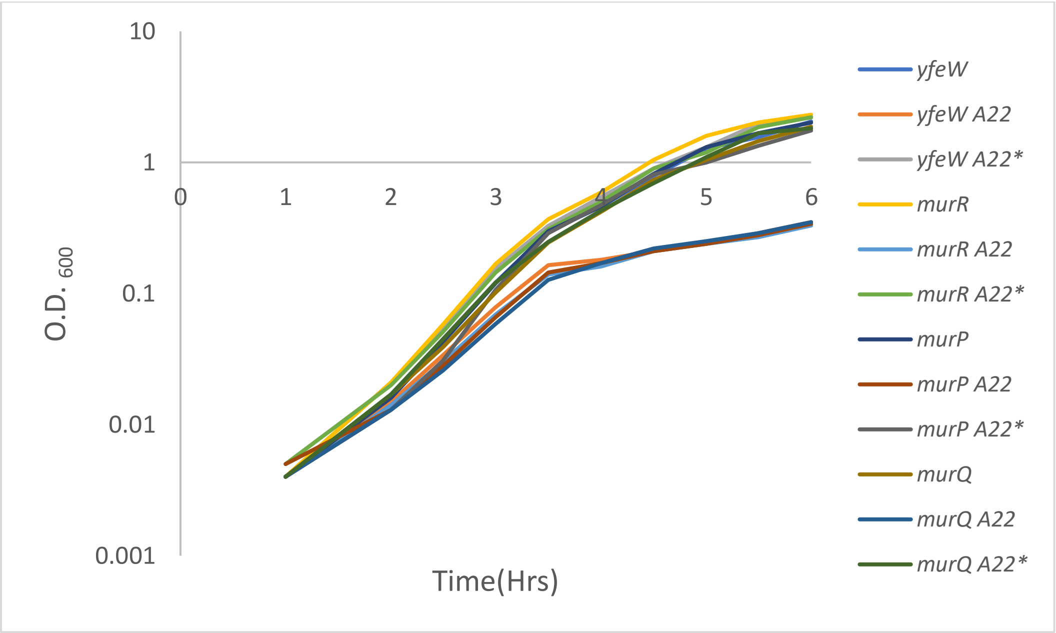

Figure S21. **Additional repetitions of potential DDGR gene growth curves.** (ab) growth curves of Δ*yfeW*, Δ*murR*, Δ*murP*, and Δ*murQ* cells grown at 37 ºC in LB without A22, A22 (10 µg/ml), and A22 added at $O.D._{600}$ ~0.1 (*).
