# Supplementary material for: Cell density-dependent antibiotic tolerance to inhibition of the elongation machinery requires fully functional PBP1B
Source: Commun Biol. 2022 Feb 3;5:107. doi: 10.1038/s42003-022-03056-x (PMC8813938; doi:10.1038/s42003-022-03056-x)
Supplement: Supplementary file 2 — Description of Additional Supplementary Files [file 42003_2022_3056_MOESM2_ESM.pdf]

Description of Additional Supplementary Files

File name: Supplementary Data 1
Description: The raw used to make the growth curves in the main figures.
